# Time-course RNASeq of *Camponotus floridanus* forager and nurse ant brains indicate links between plasticity in the biological clock and behavioral division of labor

**DOI:** 10.1186/s12864-021-08282-x

**Published:** 2022-01-15

**Authors:** Biplabendu Das, Charissa de Bekker

**Affiliations:** 1 Department of Biology, College of Sciences, University of Central Florida, Orlando, FL 32816 USA; 2 Genomics and Bioinformatics Cluster, University of Central Florida, Orlando, FL 32816 USA

**Keywords:** Carpenter ants, Behavioral division of labor, Plastic timekeeping, Diurnal rhythms, Ultradian rhythms, Time-course RNASeq

## Abstract

**Background:**

Circadian clocks allow organisms to anticipate daily fluctuations in their environment by driving rhythms in physiology and behavior. Inter-organismal differences in daily rhythms, called chronotypes, exist and can shift with age. In ants, age, caste-related behavior and chronotype appear to be linked. Brood-tending nurse ants are usually younger individuals and show “around-the-clock” activity. With age or in the absence of brood, nurses transition into foraging ants that show daily rhythms in activity. Ants can adaptively shift between these behavioral castes and caste-associated chronotypes depending on social context. We investigated how changes in daily gene expression could be contributing to such behavioral plasticity in *Camponotus floridanus* carpenter ants by combining time-course behavioral assays and RNA-Sequencing of forager and nurse brains.

**Results:**

We found that nurse brains have three times fewer 24 h oscillating genes than foragers. However, several hundred genes that oscillated every 24 h in forager brains showed robust 8 h oscillations in nurses, including the core clock genes *Period* and *Shaggy*. These differentially rhythmic genes consisted of several components of the circadian entrainment and output pathway, including genes said to be involved in regulating insect locomotory behavior. We also found that *Vitellogenin*, known to regulate division of labor in social insects, showed robust 24 h oscillations in nurse brains but not in foragers. Finally, we found significant overlap between genes differentially expressed between the two ant castes and genes that show ultradian rhythms in daily expression.

**Conclusion:**

This study provides a first look at the chronobiological differences in gene expression between forager and nurse ant brains. This endeavor allowed us to identify a putative molecular mechanism underlying plastic timekeeping: several components of the ant circadian clock and its output can seemingly oscillate at different harmonics of the circadian rhythm. We propose that such chronobiological plasticity has evolved to allow for distinct regulatory networks that underlie behavioral castes, while supporting swift caste transitions in response to colony demands. Behavioral division of labor is common among social insects. The links between chronobiological and behavioral plasticity that we found in *C. floridanus*, thus, likely represent a more general phenomenon that warrants further investigation.

**Supplementary Information:**

The online version contains supplementary material available at 10.1186/s12864-021-08282-x.

## Background

Living organisms exhibit adaptive rhythms in physiology and behavior as a way to anticipate predictable daily fluctuations in their environment [1–3]. Such daily rhythms are ubiquitous and have been discovered in both unicellular and multicellular organisms [4–9], including eusocial Hymenopterans such as ants and bees [10–16]. These rhythms are driven by endogenous molecular feedback loops that are capable of entraining to external time cues, known as Zeitgebers, which can be both abiotic (e.g., light and temperature cycles) and biotic (e.g., presence of food and predators) [17–20]. In the majority of model organisms studied thus far, light appears to be the strongest Zeitgeber [19, 21]. However, it has been suggested that in Hymenopterans with complex social behaviors, temperature cues and social environment could be more potent Zeitgebers than light [22–26]. Though, a more thorough molecular understanding of the Hymenopteran clock and its role in the social organization of insect colonies is needed to confirm this.

Our knowledge of the molecular underpinnings of the Hymenopteran clock is limited [14–16, 27–29]. This is in stark contrast with our vast molecular understanding of the circadian clock of *Drosophila melanogaster*, which has been extensively studied and is often used as a reference model for insect circadian clocks in general (reviewed in [30–32]). At the cellular level, the circadian clock consists of an autoregulatory transcription-translation feedback loop (TTFL) that requires around (circa) 24 h (dia) to complete one cycle. The circadian TTFL is considered to be an ancient timekeeping mechanism conserved in plants, fungi and animals [2, 30, 33]. In the insect model organism *Drosophila*, the TTFL consists of the activator complex CLOCK-CYCLE (BMAL1-CLOCK in mammals) that binds to and activates transcription of the repressor gene *Period* (*Per*). Upon translation in the cytoplasm, PER heterodimerizes with TIMELESS (CRYPTOCHROME in mammals), translocates into the nucleus and inhibits the CLK-CYC activator complex, thus closing the feedback loop [34, 35]. This loop is further coupled with multiple auxiliary phosphorylation-dephosphorylation cycles, that are necessary for a functional 24-h clock [34, 35]. Several kinases (e.g., Shaggy, Double-time, Nemo, Casein Kinase-2 and Protein Kinase A) and phosphatases (e.g., Protein phosphatase 1 and Protein phosphatase 2A) involved in such auxiliary cycles have been discovered in *Drosophila* (reviewed in [31]). Once entrained, the circadian clock drives daily oscillations in gene expression and protein production that in turn bring about rhythms in physiology (e.g., metabolism and immune function) and behavior (e.g., locomotion and feeding) [36].

In addition to being endogenous and entrainable, circadian clocks are also inherently plastic; the phase, amplitude and period length with which circadian processes oscillate can change with an organism’s age or social environment [37–42]. Such changes give rise to phenotypes that differ in their exact timing of activity onset relative to sunset or sunrise, known as “chronotypes” [43–46]. Social insects, which exhibit complex social organization and a decentralized division of colony labor, provide a striking example of plastic chronotypes which appear to be tightly associated with an individual’s behavioral role or caste identity within the colony [13, 15, 28, 47, 48]. In ants and bees, broadly two distinct behavioral castes emerge from division of colony labor among non-reproductive “workers”: 1) foragers that perform the majority of outside-nest tasks such as gathering food in an environment with daily cycling abiotic conditions and 2) nurses that perform inside-nest tasks, including brood care, in dark nest chambers with little to no abiotic fluctuations [49]. In most species studied so far, isolated ants and bees in a forager-like state show robust daily rhythms in activity whereas brood-tending nurses display “around-the-clock” activity patterns with no apparent rhythmicity [25, 48, 50, 51]. In honeybees, foragers coerced into tending brood will begin to show “around-the-clock” activity whereas brood-tending nurses develop robust locomotory rhythms upon removal from the colony [15, 27, 52]. Similarly, in carpenter ant workers, the presence or absence of rhythmic activity state is tightly linked with their social context and caste identity in the colony [13, 48, 53]. For example, in the carpenter ant *Camponotus rufipes*, nurses showed a rapid development of rhythmic activity patterns when isolated from the colony and placed under cycling light–dark conditions [48]. This rhythmic activity persisted under constant darkness conditions in the absence of brood [48]. Similarly, isolated individuals of the ant species *Diacamma indicum*, showed rhythmic activity under LD cycles in the absence of eggs and larvae, but transitioned to nurse-like “around-the-clock” activity in their presence [25]. As such, 24 h-rhythms in locomotory behavior appear to be regulated by an individual’s social context and behavioral role in the colony [25, 26, 48, 54]. This is in line with the finding that social cues, such as colony odor or substrate-borne vibrations, can be potent Zeitgebers in social insects and can even override photic entrainment [23, 24].

The molecular aspects of plastic timekeeping and its role in driving behavioral plasticity that gives rise to colony-wide division of labor in ants, and other social insects, are largely unexplored. Exposing the mechanisms of plastic timekeeping in ants, and how they connect to behavioral phenotypes, could be essential in our understanding of eusocial behavior and regulation of colony functioning. A first step in this direction has been made by Rodrigues-Zas and colleagues, who investigated 24 h-rhythms in gene expression in honeybee forager and nurse brains through a time-course microarray study [16]. However, this study identified only 4% of all protein coding genes as rhythmic, which seems almost certainly a vast under-representation considering the abundance of clock-controlled genes that have been found in other organisms [55–61]. No other genome-wide reports that assess daily rhythms in gene expression seem to exist for Hymenoptera despite the availability of newer high-throughput sequencing techniques and improved rhythm detection software [62, 63]. As such, a major knowledge gap regarding the inner workings of social insect clocks, and especially those of ants, remain. This greatly limits our ability to investigate how biological clocks could be interacting with social cues to produce functionally distinctive behavioral castes with their own characteristic chronotypes.

Our current study aims to address this knowledge gap by investigating rhythmic gene expression, throughout a 24 h-day, in brains of *Camponotus floridanus* nurse and forager ants. The Florida carpenter ant, *C. floridanus*, produces large colonies with several thousand workers, organized in both behavioral and morphological castes. This species is considered an urban pest [64] and is frequently used in a wide variety of social insect studies (e.g., [65–74]). To collect forager and nurse ants of *C. floridanus*, we conducted a time-course experiment on a single medium-to-large colony, kept in a complex behavioral setup that allowed us to quantify daily rhythms in colony activity and identify forager-nurse castes based on behavior. We subsequently used the brains of collected foragers and nurses for RNASeq to fulfill three primary objectives: (1) to investigate the extent of rhythmic gene expression for both castes, (2) to characterize the similarities and differences in their daily transcriptomes, and (3) to identify putative mechanisms that could allow brood-tending nurse ants, known to show arrhythmic behavior, to possess a functional timekeeping machinery. Given that we sampled ants under LD cycle, we use the term “diurnal” throughout the article to refer to 24 h-rhythms, in behavior and gene expression, since we cannot distinguish internally- and externally-driven rhythms. We found that nurse brains harbored a reduced number of diurnal genes as compared to foragers. Yet, we discovered that several genes with robust diurnal expression in forager brains were not entirely arrhythmic in nurses. Rather, these genes oscillated with 12-h and 8-h periodicities (the core clock gene *Period* being one of them). We discuss the possibility that such plasticity in clock and clock-controlled gene expression could facilitate swift nurse to forager transitions and vice-versa. Furthermore, we used functional enrichments of gene ontology annotations to identify biological processes that are seemingly under clock-control in *C. floridanus* brains, and highlight the ones enriched for genes that cycled at different periodicities in the two ant castes. Additionally, we report on genes that were expressed at vastly different levels in the brains of the two ant castes, throughout the day. The protein products of several of these differentially expressed genes have been discovered in the trophallactic fluid of *C. floridanus* [71, 75]. As such, we discuss the possibility that division of labor and the regulation of behavioral chronotypes in ant societies is trophallaxis-mediated.

## Results and Discussion

### Daily rhythms in colony behavior of *Camponotus floridanus*

**Fig. 1:**
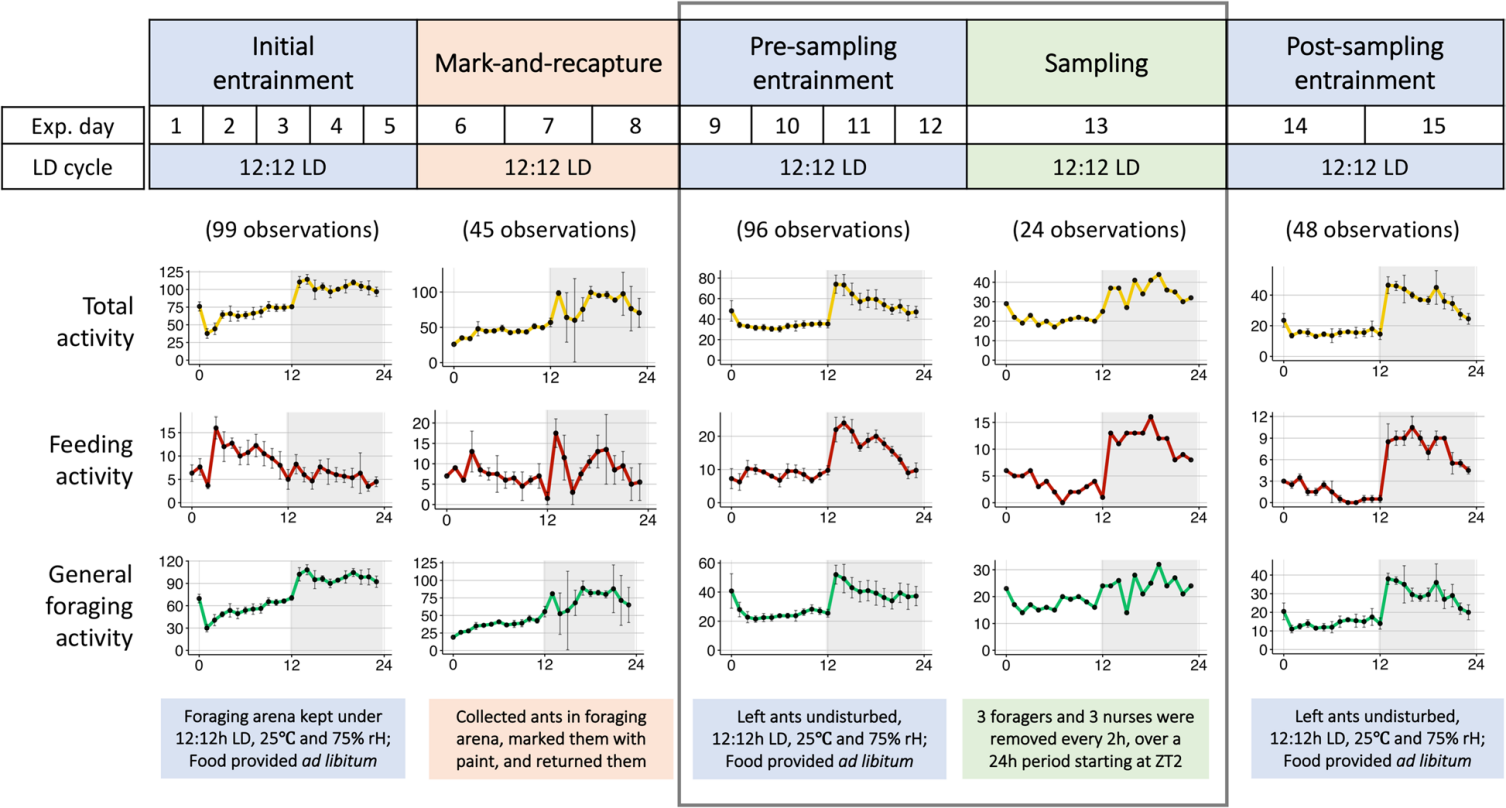
Daily rhythms in colony activity. The top panel shows the experimental timeline and the bottom graphs show the mean (± SE) daily extranidal activity of the ant colony during each phase of the experiment. During the entire experiment, the foraging arena was kept at 25ºC, 70% rH and under oscillating 12 h:12 h light–dark (LD) cycles. Undisturbed phases under light–dark cycles are shown in blue, while experimental phases of disturbance are shown in orange (mark-and-recapture of foragers) and green (sampling of ants for RNASeq). For each plot, colored lines connecting the dots represent average activity while black bars represent one standard error around the mean. The y-axis represents number of ants and the x-axis represents Zeitgeber Time (ZT) during the 12 h:12 h LD cycle. The shaded part of the plots represents the dark phase (ZT12-24). The number of ants actively feeding or present on the feeding stage is plotted as the feeding activity. The general foraging activity is the number of ants present in the foraging arena but not on the feeding stage. The total activity is the sum of feeding and foraging activity, representing the total extranidal activity of the colony at a given time. The number of observations used to calculate the mean (± SE) activity for each phase are shown in parenthesis at the top of the plots. Missing data points during ‘Initial entrainment’ and ‘Mark-and-recapture’ were due to inability to get accurate count of ants from video frames and a recording failure, respectively

We collected forager and nurse ants from a single *C. floridanus* colony, preventing potential inter-colony differences in timing of foraging from obscuring the inter-caste differences in gene expression that we aimed to measure. *Camponotus floridanus* is known to be largely nocturnal both in nature (personal field observations, [64]) and in the lab [69, 70]. Despite this knowledge, we first had to entrain and quantify the colony-level behavioral rhythms of *C. floridanus* to be able to reliably investigate the daily gene expression underlying their seemingly clock-regulated behavioral activity. Therefore, we recorded extranidal visits of a large *C. floridanus* colony, housed in a darkened nest, that we attached to a foraging arena subjected to a 12 h:12 h LD cycle (see Methods section for more details). Subsequently, we counted the number of foraging ants throughout the day that were actively feeding or present on the feeding stage (Fig. 1, “feeding” or feeding activity) as well as in the remainder of the foraging arena (Fig. 1, “foraging” or general foraging activity). We defined the colony’s total foraging activity (Fig. 1, “Total activity”) as the sum of feeding and foraging at any given time. The first signs of initial colony entrainment were visible through the early establishment of a day-night rhythm in foraging (Fig. 1, Day 1–5). In the following 3 days, we performed mark-and-recapture to identify ants of the foraging caste. During this time the foraging rhythm was somewhat less pronounced but managed to stay intact (Fig. 1, Day 6–8). From Day 9 onwards, both feeding and foraging showed pronounced day-night rhythms that persisted during and beyond the sampling day (Fig. 1, Day 9–15). These day-night rhythms followed a consistent pattern with increased foraging activity during the night-time as compared to the daytime, similar to previously reported locomotory rhythms of isolated *C. floridanus* ants [70]. Thus, based on extranidal activity of the foraging caste, the colony established robust nocturnal activity rhythms as it would in nature by entraining to the light Zeitgeber we provided.

**Fig. 2:**
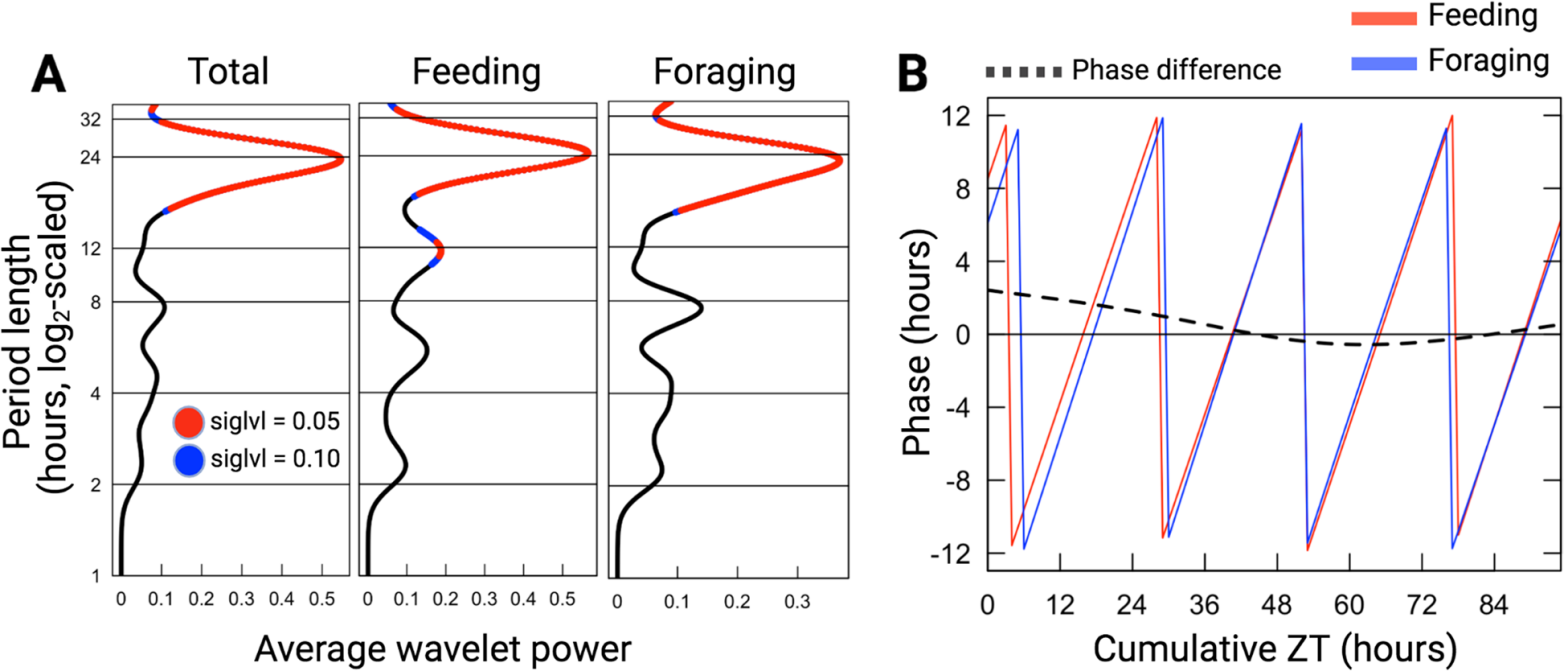
Wavelet analysis of feeding and general-foraging activity rhythms*.* **A** Dominant periods identified using wavelet decomposition of each activity profile during the continued entrainment phase (Day 9–12). The x-axis shows the average wavelet power for different period lengths. The y-axis shows the period length (log2-scaled) in hours. Significant period lengths (siglvl < 0.05) are shown in red, and the peak indicates the dominant period having the most power (around 24 h for all three activity profiles, and an additional 12 h peak observed in feeding bouts); **B** The plot shows the phase (on the y-axis) of feeding bouts (Feeding) and general foraging activity (Foraging) during continued entrainment. The dotted line indicates the phase difference of feeding over foraging during the same time period. Positive phase difference indicates that feeding leads foraging. The x-axis shows the cumulative hours passed since disturbance due to mark-and-recapture (Cumulative ZT)

To further characterize the behavioral rhythms in the entrained *C. floridanus* colony and to investigate the potential behavioral effects of the disturbance introduced by the mark-recapture, we performed wavelet analyses [76] on the foraging data collected during the four-day period just after mark-recapture and prior to sampling (Fig. 1, Day 9–12). *Camponotus floridanus* ants of the foraging caste showed significant 24 h-rhythms in feeding and foraging activity (Fig. 2A). Average wavelet powers indicated that both feeding and foraging activity profiles comprised of significant waveforms with a period length close to 24 h (Fig. 2A). Neither feeding nor foraging activity peaked exactly at lights-off (ZT12). Rather, we noticed a sharp increase in both activities about an hour later (~ ZT13) (Fig. 1, Day 9–12). After peaking around ZT13-15, both feeding and foraging activity continued to decrease throughout the night and reached their daily minimum shortly after lights were turned on (ZT2-4) (Fig. 1, Day 9–12). In Central Florida (the location of colony collection), dusk lasts for 84 (± 5) minutes after sunset (Additional File 1A, data retrieved from www.timeanddate.com). In our experimental setup, we chose an abrupt light–dark transition, and hence, did not provide twilight cues. Therefore, the stark increase in extranidal activity within an hour post lights-off, could be indicating an endogenous dusk-entrainment in colony foraging activity. Taken together, the colony activity rhythms that we observed for *C. floridanus* – 24 h-rhythmic and predominantly nocturnal, with a dusk-phase – largely resembled previously reported activity patterns [70]. This indicates that the experimental setup that we designed allowed us to collect daily gene expression data related to expected ant daily activity patterns.

In addition to the dominant 24 h-rhythm, we detected a significant circa-12 h rhythm in feeding activity (Fig. 2A). Inspection of feeding power-spectra over the four days of continued-entrainment revealed that, while the 24 h-rhythm was sustained throughout, the 12 h rhythm was only significantly present during the first 36 h post disturbance. Within this 36 h time-period, integration of the 12 h and 24 h waveforms improved fit (Additional File 1B). A possible explanation for the presence of this short-lived 12 h activity rhythm could be that it played a role in catching up with feeding needs of the colony in the initial hours after disturbance. The removal of foragers during mark-recapture most likely desynchronized the colony’s daily feeding pattern and might explain the lack of a clear diurnal activity in colony feeding and a diminished overall 24 h foraging pattern during the mark-recapture period (Fig. 1; Day 6–8). As such, we enquired if the circa-12 h rhythm in feeding could be important to re-establish a rhythmic colony feeding behavior that is synchronous to the colony’s foraging activity. To this end, we calculated the phase difference of the 24 h-wavelets for feeding-over-foraging throughout the four days post mark-recapture (Fig. 2B). At the start of pre-sampling entrainment (i.e., right after disturbance by mark-recapture), feeding was found to lead general foraging by more than two hours. Approximately 36 h into the pre-sampling entrainment period, the phase difference reduced to zero; 24 h-rhythms in feeding and foraging aligned. Subsequently, the phase difference between feeding and foraging remained close to zero (Fig. 2B). This data suggests that, indeed, after three consecutive nights of disrupted feeding, the colony attempted to get back on track through a short initial phase shift between feeding and foraging. Once synchrony between the phases of the two activities was restored, it was maintained. The intermittent 12 h feeding peaks observed during the first 36 h after mark-recapture (Additional File 1B) likely contributed to restoring this synchrony.

### General patterns of gene expression in *C. floridanus* brain tissue

After twelve days of LD entrainment, we collected three *C. floridanus* foragers and nurses from the colony every 2 h, over a 24-h period (Fig. 1, Day 13). Individuals that were collected in the foraging arena and paint-marked as part of our mark-recapture efforts were collected as foragers. Unmarked individuals that interacted with the brood inside the dark nest chambers were collected as nurses. We subsequently used RNA-Seq to obtain the transcriptome profiles of forager and nurse brain tissue.

Of the 13,808 protein coding genes annotated in the *C. floridanus* genome [72], 8% (1130 genes) were not expressed (i.e., FPKM: 0) and 19% (2640 genes) were only lowly expressed in forager and nurse brains (i.e., 0 < FPKM ≤ 1) throughout the day (Additional File 2, sheet 1). The majority of genes involved in olfactory and gustatory functions in *C. floridanus* were among these lowly expressed genes (93% of 363 genes involved in sensory perception of smell and 73% of 26 genes involved in sensory perception of taste) (Additional File 2, sheet 2). Notably, majority of the genes involved in hormone activity (69% of 16), metallopeptidase activity (86% of 110), and nucleotide binding (85% of 27) were found to be enriched among the genes that showed either no or low expression (Additional File 2, sheet 2). The clear overrepresentation of certain gene functions among genes that were either lowly or not expressed necessitated the use of a reduced background gene set for subsequent enrichment analyses that consists of only those genes that were actually expressed. This, to avoid obtaining gene function enrichments that merely reflect brain tissue specific gene expression. We classified genes to be expressed in *C. floridanus* brains if mRNA levels were greater than 1 FPKM for at least one time point, for either behavioral caste, during the 24 h sampling period.

We found 71% (i.e., 9843 genes in foragers and 9872 genes in nurses, Additional File 2, sheet 3) of all protein coding genes to be expressed in ant brains. Of these genes, 166 were uniquely expressed in the forager brains and 195 in nurses. One *odorant receptor 4-like*, two *odorant receptor 13a-like*, and two other uncharacterized odorant receptor genes were among those uniquely expressed in forager brains, along with several proteases. In addition to significant enrichments in olfaction and proteolysis-related biological processes, uniquely expressed genes in foragers were also enriched in the cellular component nucleosome and included several histone-related genes (Additional File 2, sheet 4). In comparison, genes uniquely expressed in nurses were enriched in redox and lipid metabolic processes and included several putative *cytochrome P450* and *lipase 3-like* genes (Additional File 2, sheet 4). This is in line with the canonical behavioral and physiological differences that characterize foragers and nurses in a social insect colony. A fine-tuned olfactory and gustatory repertoire in foragers is essential for trail-following and other general foraging tasks. In contrast, metabolic processes have been previously found to be upregulated in intranidal nurse workers that are usually tasked with larval feeding and brood care [77]. This indicates that the expression data that we obtained is likely a good representation of the gene expression profiles that are characteristic for both castes.

### Diurnal rhythms in gene expression

**Fig. 3:**
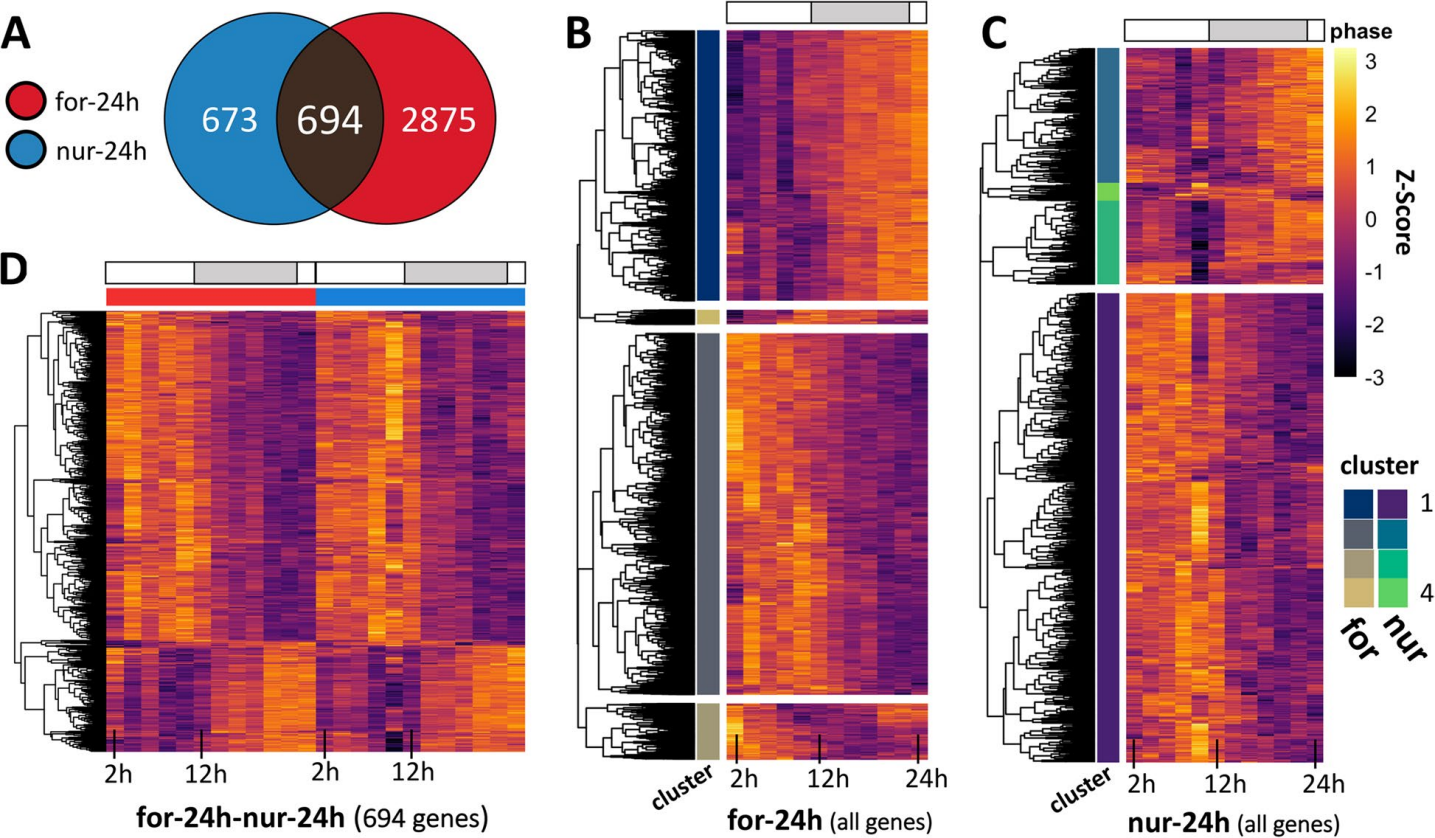
Diurnal rhythms of gene expression in the ant brain **A** Venn-diagram showing the number of genes significantly oscillating every 24 h in forager (for-24 h) and nurse (nur-24 h) brains. The heatmaps show the daily expression (z-score) patterns of all identified 24 h-oscillating genes in **B** foragers (for-24 h), **C** nurses (nur-24 h), and **D** both foragers and nurses (for-24 h-nur-24 h). Each row represents a single gene and each column represents the Zeitgeber Time (ZT) at which the sample was collected, shown in chronological order from left to right (from ZT2 to ZT24, every 2 h). The grey bar above the heatmaps runs from ZT12 to ZT24 and indicates the time during the light–dark cycle in which lights were off. Both for-24 h and nur-24 h genes were hierarchically clustered into four clusters. The cluster identity of each gene is indicated in the cluster annotation column

We used the non-parametric algorithm empirical JTK Cycle (eJTK) [78, 79] to detect diurnal (24 h) rhythms in gene expression in forager and nurse ant brains. Of the 10,038 genes expressed in *C. floridanus* brains, 42% (i.e., 4242 genes) had significant diurnal expression patterns in either foragers or nurses (Additional File 3, sheet 1 and 2). The number of putative diurnal genes in foragers was almost three times higher (i.e., 3569 genes; Fig. 3A and B, indicated with “for-24 h”) as compared to nurses (i.e., 1367 genes; Fig. 3A and C, indicated with “nur-24 h”). Only 16% of all identified diurnal genes cycled in both behavioral castes with a 24 h rhythm (i.e., 694 genes; Fig. 3A and D, indicated with “for-24 h-nur-24 h”), which represents half of all the diurnal genes that we identified in nurses. The reduced number of diurnal genes in nurses is consistent with the previous time-course microarray study done in honeybees (541 probes in forager bees and 160 probes in nurses were found to have 24 h-rhythms) [16]. This suggests that a reduced circadian control at the level of gene expression in “around-the-clock” active nurses as compared to rhythmically active foragers is likely a convergent pattern since bees and ants have evolved eusociality independently.

After identifying putative 24 h cycling genes in the two behavioral groups, we asked if they contained functional annotations with coordinated temporal peak activity (i.e., are certain biological functions “day-peaking” or “night-peaking”) and if such a temporal division of clock-controlled processes can be found in both foragers and nurses. To answer these questions, we used an agglomerative hierarchical clustering framework to group the diurnal genes in foragers and nurses into four gene clusters (Additional File 3, sheet 3 and 4). We followed this analysis by identifying significantly enriched gene ontology (GO) terms for each identified gene cluster.

The choice of four clusters was aimed to demarcate, if possible, potential day-, night-, dawn-, and dusk-peaking genes. Using this method, we identified that more than half of all diurnal genes in foragers showed a peak activity during early-to-mid daytime (1916 genes, Fig. 3B, for-24h_Cluster2). The majority of the remaining genes showed peak expression activity around late night-time (1417 genes, Fig. 3B, for-24h_Cluster1). Additionally, one of the two smaller clusters of genes that cycled with a 24 h rhythm in foragers (74 genes, Fig. 3B, for-24h_Cluster4) appeared to peak at dusk with an acrophase around ZT12-14. Among these dusk-peaking genes we identified the putative insect melatonin receptor *trapped in endoderm* (*tre1; MTNR1a* in mammals), which has been reported to be central to the dusk/dawn entrainment pathway in humans (Table 1) [80–82]. The genes in nurse brains that showed 24 h rhythms also primarily clustered into two groups – day-peaking (909 genes, Fig. 3C, nur-24h_Cluster1) and night-peaking genes (261 genes, Fig. 3C, nur-24h_Cluster2) – with only a few genes in the remaining two clusters (Cluster 3, 162 genes; Cluster 4, 35 genes).

**Table 1:** Clock components of *Camponotus floridanus* and their gene expression patterns in forager and nurse brains. The table below lists the *C. floridanus* homologs of several *Drosophila* core-clock, clock-modulator and clock-output genes. The periodicity (tau) of rhythmic gene expression in the brain, if any, is indicated for both foragers and nurses. The one-to-one ortholog of the identified *C. floridanus* gene in mammals and honeybees is also provided. A dash in the periodicity column indicates that no significant daily rhythms were detected for the *C. floridanus* gene, whereas a dash in the ortholog columns indicates that no one-to-one orthologs of the *C. floridanus* gene was detected. The genes that show differential rhythmicity, oscillating at two distinct periodicities, in the two ant castes are shown in bold

Homologs of key insect clock components present in *Camponotus floridanus* (*Cflo*)			Periodicity (tau) of gene expression		One-to-one ortholog of the *Cflo* gene in	
*Drosophila gene*	*Cflo homolog*	*Function*	*Forager*	*Nurse*	*mice or* *humans*	*honeybees*
*Clock*	LOC105257275	core-clock	12 h	-	*Npas2*	*Clock*
***Period***	**LOC105256454**	**core-clock**	**24 h**	**8 h**	***-***	***Per***
*Vrille*	LOC105252510	core-clock	8 h	-	-	*Ataxin-2* homolog
*Double-time*	LOC105255207	modulator	24 h	-	*Ck1d/e*	*Ck1*
*Casein kinase 2 alpha*	LOC105256631	modulator	24 h	24 h	*Ck2a*	*Ck2a*
***Shaggy***	**LOC105258655**	**modulator**	**24 h**	**8 h**	***Gsk3b***	***Sgg***
*Nemo*	LOC105248529	modulator	24 h	-	*Nlk*	*Nlk2*
***Protein phophatase 1b***	**LOC105251553**	**modulator**	**24 h**	**8 h**	***Pp1b***	***Pp1b***
***Pp1***	**LOC105250191**	**modulator**	**24 h**	**8 h**	***-***	***-***
*Rhodopsin*	LOC105252466	modulator	24 h	24 h	*Opn4*	*Lop1*
*mAchR*	LOC105253861	output	24 h	-	-	*mAchR*
***DopEcR***	**LOC105257836**	**output**	**24 h**	**8 h**	***Gpr52***	***DopEcR***
*Pigment dispersing factor*	LOC105256952	output	24 h	-	-	*Pdf*
*Pdf receptor*	LOC105252917	output	24 h	-	-	*Pdfr*
*Protein kinase A*	LOC105249574	output	24 h	-	*Prkaca/b*	*Pka*
*Lark*	LOC105259208	output	24 h	24 h	*Rbm4*	*Lark*
***Protein kinase C***	**LOC105255087**	**output**	**24 h**	**8 h**	***Prkci***	***Pkc***
*Trapped in endoderm 1*	LOC105250997	output	24 h	-	*MT1*	*Tre1*
*Slowpoke*	LOC105258647	output	24 h	-	*Slo*	*Kcnma1*

Despite the relatively smaller number of day-peaking and night-peaking diurnal genes in nurses, we found functional enrichments comparable to those found in foragers. The night-peaking gene clusters in foragers and nurses were both enriched in genes with the annotated GO terms: regulation of transcription (DNA-templated), signal transduction and protein phosphorylation (Additional File 3, sheet 5). This indicates that a significant number of night-peaking diurnal genes in nurse and forager brains seem to be involved in cell–cell communication, gene expression, and protein modification. The day-peaking diurnal gene clusters in both behavioral groups were enriched for genes involved in metabolism (glycosylphosphatidylinositol (GPI) anchor biosynthesis) (Additional File 3, sheet 5). In addition, the diurnal gene clusters in foragers were enriched for multiple other biological processes that were not found to be enriched in nurses. The day-peaking genes in foragers were enriched for GO terms that concerned response to stress, as well as tRNA, mRNA and translational processes, and terms involved in post protein processing such as folding and transport (Additional File 3, sheet 5). Night-peaking genes in foragers were additionally enriched in terms such as regulation of transcription by RNA polymerase II, multicellular organism development, protein homooligomerization, microtubule-based movement, G protein-coupled receptor signaling pathway, and ion transmembrane transport (Additional File 3, sheet 5). This temporal segregation of clock-controlled processes in foragers appears to be in line with findings from previous studies done on the fungus *Neurospora crassa*, mammals and flies [57, 59, 83]. However, while the daily transcriptome of rhythmic foragers revealed the expected temporal separation, nurse gene expression showed a much more limited temporal organization. This provides further evidence for a reduced diurnal control in “around-the-clock” active nurses as compared to rhythmically active foragers.

The question that remains is if the shared functional enrichments among the 24 h rhythmic genes in both ant castes encompass the same exact genes or if they are different but with similar functions. To answer this question, we analyzed the functional annotations of the 694 diurnal genes that were shared between foragers and nurses. Hierarchical clustering revealed that these genes predominantly peaked during the daytime (Fig. 3D) and that the shared day-peaking genes were significantly enriched in the functional annotation GPI anchor biosynthesis (genes *Pig-b, Pig-c, Pig-g, Pig-m,* and *Mppe*) (Additional File 3, sheet 5). However, the relatively smaller set of shared night-peaking diurnal genes was not enriched in any functional annotations. Using a Fisher exact to test for significant overlap between genesets, we found that the night-peaking activity of regulation of transcription (DNA-templated) (Odds-ratio: 0.55; *p*-value: 0.89), signal transduction (Odds-ratio: 7.48; *p*-value: 0.06) and protein phosphorylation (Odds-ratio: 2.12; *p*-value: 0.21) are mostly due to different sets of diurnal genes in foragers and nurses, but with similar functions. In contrast, GPI anchor biosynthesis activity appears to be driven by the same day-peaking diurnal genes in both ant castes.

**Fig. 4:**
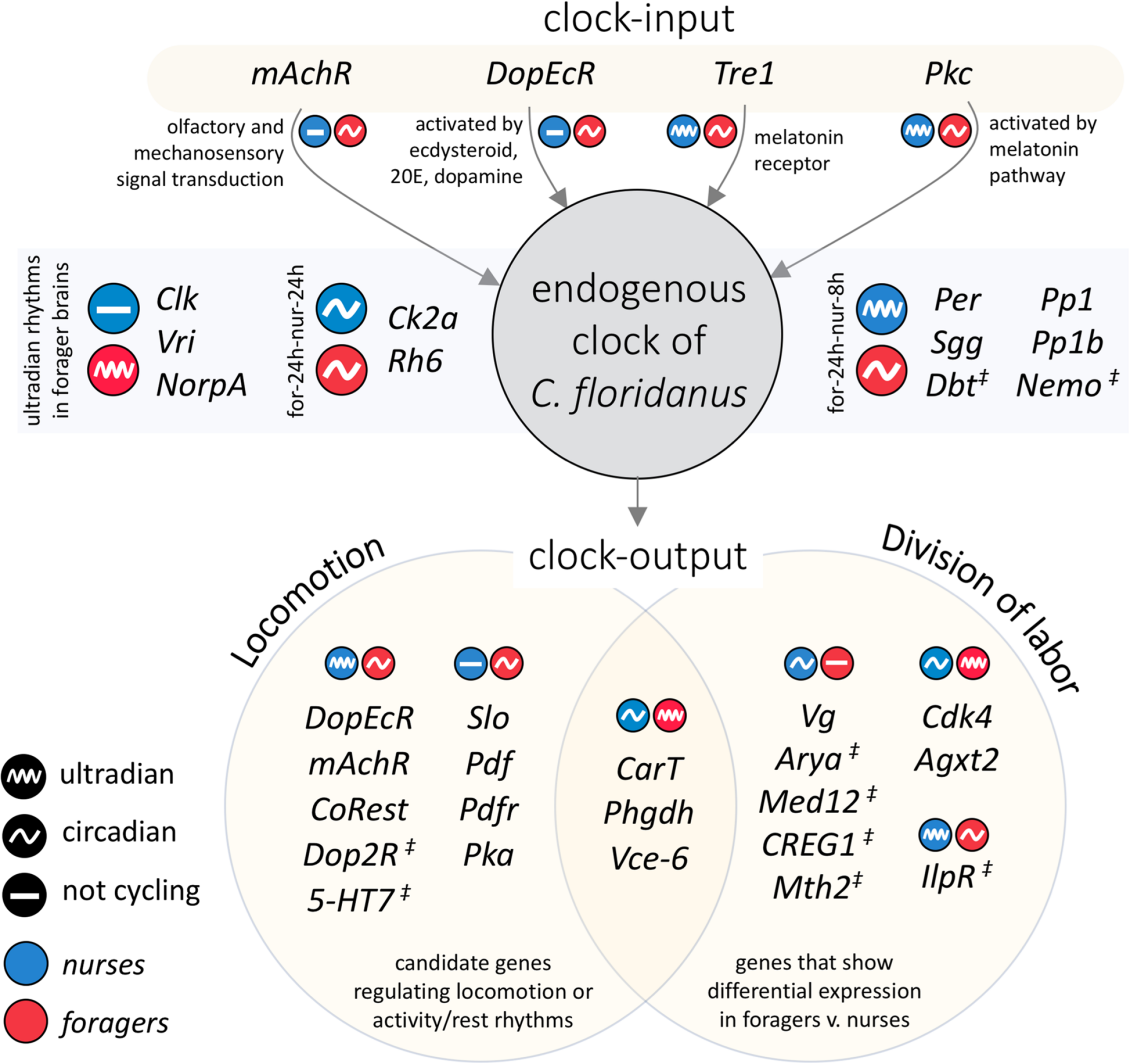
Potential links between chronobiological plasticity and behavioral division of labor in *C. floridanus***.** The infographic shows differences in rhythmic expression in forager and nurse brains for several genes involved in entrainment of the endogenous clock (clock-input), proper functioning of the endogenous clock, and the clock-controlled pathways (clock-output) that likely regulate locomotion and division of labor in ants. The symbol “‡” indicates that gene expression for that gene shows a trend of rhythmic expression in one of the ant castes (Additional File 5) but was not significant (*p* ≥ 0.05). Ultradian rhythms include both 8 h and 12 h oscillations. The following genes have been abbreviated in the figure but not in the text: *Venom-carboxylesterase-6* (*Vce-6*), *Arylphorin-subunit-alpha* (*Arya*)

The molecular underpinnings of timekeeping in nurse ants, and other animals with “around-the-clock” activity, is still elusive [14, 16, 84]. To find candidate genes presumably involved in daily timekeeping in *C. floridanus* nurses, we queried the diurnal genes that they shared with foragers for known components of the insect clock (Additional File 4). The shared day-peaking gene cluster contained one known clock output gene (i.e., *Lark*) and two genes known to modulate the circadian clock – *Casein kinase 2 alpha* (*Ck2a*) and the light-dependent *Rhodopsin* (*Rh6*; orthologous to mammalian *Opn4*) (Table 1, Fig. 4). Along with other members of the opsin gene family, the *Rh6* gene in *Drosophila* has been shown to also have light-independent functions in thermosensation (in larvae) and hearing (in adults) [85, 86]. The auditory role of opsins, likely mediated by mechanotransduction [87], could be especially relevant for circadian entrainment in social insects. Ants and bees are known to use vibroacoustic means such as “drumming” behavior (i.e., vibrations produced by tapping the nest substrate with their head and gaster) to communicate within dark nest chambers [88–91]. Moreover, there is recent evidence that substrate-borne vibrations are potent social Zeitgebers capable of entraining the circadian clock of newly emerged honey bees housed in the dark [24]. These substrate-borne vibrations could potentially play a similar role in the social entrainment of nurse ants through the light-independent involvement of a rhodopsin-mediated mechanosensory pathway [87], while extranidal foragers might also make use of its light-dependent functions.

In addition to *Rh6,* the kinase *Ck2a* showed robust 24 h rhythms and a near-perfect alignment in gene expression between the behavioral groups (Additional File 3, Fig. 4). *Ck2a* encodes the catalytic subunit of the circadian protein, Casein Kinase 2 (CK2). In *Drosophila*, CK2 appears to regulate rhythmic behavior by phosphorylating the core clock proteins PERIOD (PER) and TIMELESS (TIM) [92–95]. This CK2-mediated phosphorylation is perceived as a rate-limiting step in the circadian clock, important for a functional 24 h transcription-translation feedback loop [95]. The central role of CK2 in regulating the endogenous clock in other organisms suggests a potential role of *Ck2a* in maintaining a functional 24 h oscillator in both, “around-the-clock” active nurses and rhythmically active foragers. However, other homologs of genes encoding core clock proteins, such as PER, were not present among the diurnal genes that were shared between foragers and nurses (Table 1, Additional File 3).

### Ultradian rhythms in gene expression

**Fig. 5:**
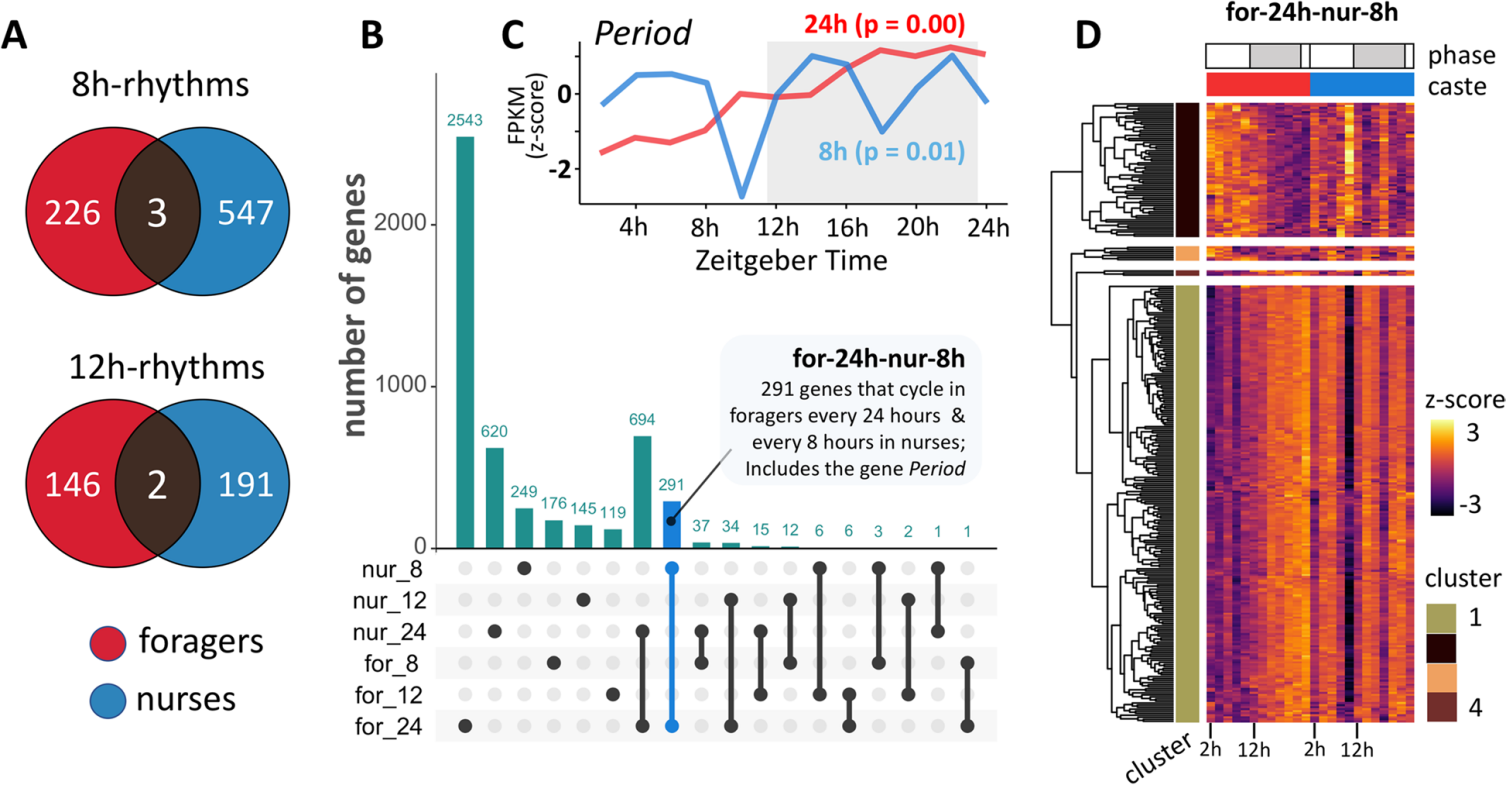
Ultradian rhythms and caste-associated differential rhythmicity in gene expression**. A** Venn-diagrams showing the number of genes with significant ultradian expression in the ant brain, oscillating every 8-h (8 h-rhythms) and 12-h (12 h-rhythms); **B** Upset plot showing the number of genes uniquely expressed in, and shared between, diurnal (24 h) and ultradian (8 h and 12 h) gene sets. Each bar represents a unique intersection between the six diurnal and ultradian genesets (e.g., for-24: 24 h-oscillating genes in foragers, nur-12: 12 h-oscillating genes in nurses, etc.). A gene is binned only once, and as such, belongs to only one intersection. Dark circles indicate the gene sets that are part of a particular intersection. For example, the first circle indicates that there are 2543 genes that are uniquely cycling in foragers with a 24 h period (for-24). Similarly, the blue bar indicates that there are 291 genes that have a significantly diurnal expression in foragers but cycle every 8-h in nurses (for-24 h-nur-8 h); **C** Caste-associated differential rhythmicity in the expression of the core clock gene *Period* is shown. The expression of *Per* cycles every 24-h in forager brains (red) and every 8-h in nurses (blue); *p*-values obtained from eJTK are provided in parenthesis. The Zeitgeber Time is indicated on the x-axis, while the y-axis shows the normalized (Z-score) gene expression. The dark phase of the 12 h:12 h light–dark cycle is represented in grey (dark phase begins at ZT12); **D** Heatmap showing the daily expression of all genes in the for-24 h-nur-8 h geneset, for nurses and foragers. Caste identity is indicated above the heatmap as a column annotation (red-foragers and blue-nurses). The for-24 h-nur-8 h geneset was clustered into four groups, and the cluster identity of each gene is indicated as row annotations (“cluster”). The majority of 8 h-cycling genes in nurses, including the *Per* gene, belong to Cluster 1 and show a night-time peak in forager heads

“Ultradian rhythms” in gene expression refer to significantly oscillating expression patterns around the second and third harmonic of 24 h-rhythms (i.e., genes cycling with periodicities of 12 h and 8 h, respectively). Such rhythms can be found in a wide range of species [96–102], and examples in which organisms switch from diurnal to ultradian gene expression due to changes in environmental circumstances have been reported [103]. When we visually inspected the expression of several genes that exhibited diurnal rhythmicity in foragers but not in nurses, we noticed that the expression of multiple such genes in nurses was relatively dampened but seemed to oscillate at a frequency higher than 24 h. As such, we used eJTK to detect if any genes were expressed with significant ultradian rhythms (Additional File 6). We identified a comparable number of genes that cycled with a 12 h period in forager and nurse brains (i.e., 148 and 193, respectively), and 2 genes that showed 12 h period in both castes (Fig. 5A). In foragers, the core-clock gene *Clock* (*Clk*) was present among the 12 h oscillating genes (Table 1, Fig. 4). However, we did not detect diurnal or ultradian rhythmicity in *Clk* expression in nurses (Table 1). As for genes that oscillated with a robust 8 h rhythm, we discovered 229 such genes in forager brains and about twice as many (550 genes) in nurses. Only three genes showed an 8 h cycling pattern in both behavioral castes (Fig. 5A).

Having identified ultradian rhythms in gene expression, we asked if genes that oscillated in a diurnal manner in forager brains, but not in nurses, were cycling in an ultradian manner in nurses. Indeed, we found that 325 (out of 2875) genes that cycled every 24 h in foragers were not arrhythmic in nurses but differentially rhythmic genes (DRGs) that showed robust 8 h (291 genes) or 12 h (34 genes) rhythms (“for-24 h-nur-8 h” and “for-24 h-nur-12 h”, respectively; Fig. 5B). Remarkably, several components of the insect clock were among the 291 DRGs that cycled every 24 h in foragers and every 8 h in nurses: *Period* (*Per*)*, Shaggy* (*Sgg*; *Gsk3b* in mammals)*, Protein phosphatase 1b* (*Pp1b*)*,* and *Protein phosphatase 1 at 13C* (*Pp1-13c* or *Pp1*) (Fig. 4, Table 1). This suggests that gene expression in nurse ant brains is, perhaps, not as arrhythmic as previously reported [28]. Instead, certain clock components in nurses seem to be cycling at a different harmonic compared to foragers, which could be partly facilitating the swift behavioral caste changes between foragers and nurses that have been observed in other studies [25, 48, 104]. As such, we continued our investigation into the genes that cycled every 24 h in foragers and every 8 h in nurses by asking if these DRGs play putative functional roles in regulating known clock-controlled processes as well as behavioral plasticity in ants.

### Plasticity of rhythmic gene expression in ant brains

In *Drosophila*, the circadian clock regulates daily rhythms in transcription via rhythmic binding of CLK and RNA Polymerase (Pol) II to the promoters of clock genes including *Per*, *Doubletime (Dbt; Ck1* in mammals*)* and *Shaggy (Sgg*, *Gsk3b* in mammals*)* [36, 105]. The kinase SGG regulates nuclear accumulation of the PER/TIM repressor complex [95, 106, 107], whereas DBT regulates its stability [108–110]. In addition to DBT, several other kinases (e.g., NEMO, CK2, and PKA) [108, 111–113] and a few phosphatases (e.g., PP1 and PP2a) [114, 115] have been identified as regulators of PER and PER/TIM stability in *Drosophila*. In the fruit fly *Drosophila,* the expression of *Per* and several other clock and clock-controlled genes peak during the night-time [105]. Similar to *Drosophila,* we observed a night-time peak in *Per* expression for *C. floridanus* foragers, which is also consistent with previous findings in fire ants and honeybees [14, 104]. Additionally, the phase of diurnal *Per* expression in *C. floridanus* foragers is consistent with the phase of oscillating PER abundance previously reported for the species [70]. For instance, the expression of *Per* and its protein product, both, peak at lights on (ZT24/ZT0). This is followed by a sharp decrease of *Per* at ZT2 which could explain, in part, the gradual decline in PER abundance during the day-time that has been reported by Kay and colleagues [70].

In our study, the daily changes in the expression of *Sgg*, *Dbt*, *Nemo*, *Pp1b* and *Pp1* mirrored the differentially rhythmic expression patterns of *Per* in the two ant castes (Fig. 5C, Table 1). Even though the 8 h rhythms of *Dbt* (*p* = 0.11) and *Nemo* (*p* = 0.11) in nurse brains were not statistically significant, their expression patterns showed a strong phase coherence with *Per* (Additional File 5). Having core clock components that simply cycle at a different harmonic, versus not showing any rhythmicity at all, could indeed explain the ability of “around-the-clock” nurses to rapidly develop forager-like rhythmic activity, in behavior and gene expression, when their social context changes [25, 48, 104]. Furthermore, hierarchical clustering of the DRGs that cycled every 24 h in foragers and every 8 h in nurses revealed that most of these DRGs clustered with *Per* (i.e., largely in-phase with the expression pattern of *Per* in foragers and nurses) (Fig. 5D, Additional File 7, sheet 1). Therefore, we hypothesized that the DRG-cluster in nurses that oscillated every 8 h with a phase similar to *Period* would be enriched for some of the same biological processes performed by 24 h cycling genes in foragers discussed above. Indeed, we found that the *Per*-like DRG-cluster was significantly enriched in functional annotations that we also identified in the night-peaking diurnal gene cluster of foragers; the GO terms: transcriptional regulation (DNA-templated), transcriptional regulation by RNA Pol II, protein phosphorylation and GPCR signal transduction (Additional File 7, sheet 2).

Moreover, the *Per*-like DRG cluster contained the muscarinic acetylcholine receptor gene *mAchR* and the insect dopamine/ecdysteroid receptor *DopEcR*, which have both been found to be clock-controlled in *Drosophila* [57, 116, 117]. The *mAchR* gene has a putative role in olfactory and mechanosensory signal transduction [118, 119]. Therefore, its differential clock-controlled regulation in foragers and nurses could be contributing to caste-specific behavioral phenotypes (Fig. 4). The same could be true for *DopEcR*, which modulates insect behavior by responding to dopamine, ecdysone and 20-hydroxyedysone [120–123]. In fact, dopamine is a known regulator of foraging activity in ants (reviewed in [124, 125]) and dopamine signaling has been found to be important in entraining the insect circadian clock as well as mediating clock-controlled behavioral phenotypes such as locomotion [126–128]. Studies in mammals suggest that certain dopaminergic oscillators are highly tunable and capable of generating 12 rhythms in locomotor activity, independent of the circadian clock, and this independent 12 h-clock coordinates metabolic and stress rhythms [129, 130]. Although we have not yet identified any biological oscillator that produces 8 h rhythms, such ultradian rhythms in gene expression has been found in both fungi [96] and animals [131]. Our finding that a set of genes, enriched for several biological processes, that oscillate in a diurnal manner in forager brains can switch to ultradian oscillations in nurses suggests that mechanistic links between chronobiological and behavioral plasticity in ants exist (Fig. 4).

It is not clear if the 8 h rhythms in ant brain gene expression are endogenously produced or socially regulated, and what the functional aspects of such rhythms are, if any. However, the social insect literature does point to one likely role for the ability of nurses to track 8 h periods: brood translocation. Workers of the carpenter ant species *Camponotus mus* have been observed to show daily rhythms in brood translocation behavior to move their brood between different temperature conditions. The measured time between the two daily brood translocations was exactly 8 h [11, 132, 133]. This suggests that the 24 h rhythm in thermal preference in *C. mus* nurses could be coupled with an 8 h oscillator that drives the observed daily timing of temperature-dependent brood translocation. Brood translocation is important for larval development, and hence, has implications for colony fitness [12]. As such, 8 h rhythms in behavioral outputs could have important adaptive functions. To begin to understand the potential roles for ultradian rhythms in the functioning of ant colonies, behavioral and molecular studies aimed at linking 8 h transcriptional rhythms and brood translocation could provide a good first step.

### Plasticity in behavioral output pathways

In flies, rhythmic activity patterns in total darkness have been related to the signaling pathway mediated by the neuropeptide Pigment Dispersing Factor (PDF) [36, 134–137]. PDF binds to the PDF receptor (PDFR) and triggers a signal transduction that increases cAMP levels and activates the protein kinase PKA [113]. A deficiency in PKA resulted in loss of fly locomotory rhythms even when *Per* oscillation was intact [138]. Moreover, PDF plays a central role in circadian timekeeping by mediating light input to the circadian clock neurons in the brain, coordinating pacemaker interactions among neurons, regulating the amplitude, period, and phase of 24 h-oscillations, and mediating output from the clock to other parts in the central brain [139–147]. Neurons that express PDF are present in the *C. floridanus* brain as well and could be mediating time-of-day information to brain regions involved in activity rhythms [70, 148–150]. In line with this, we found robust diurnal rhythms in *Pdf, Pdfr* and *Pka* gene expression in the brains of *C. floridanus* foragers (Fig. 4, Table 1). However, nurse ants, which generally reside in dark nest chambers and demonstrate a lack of 24 h-rhythms in locomotion, did not exhibit diurnal nor ultradian rhythmicity in *Pdf*, *Pdfr* and *Pka* expression (Fig. 4, Table 1). The absence of locomotory rhythms in nurse ants could, thus, also be the result of a non-oscillatory PDF signaling pathway.

### Links between division of labor and chronobiological plasticity

**Fig. 6:**
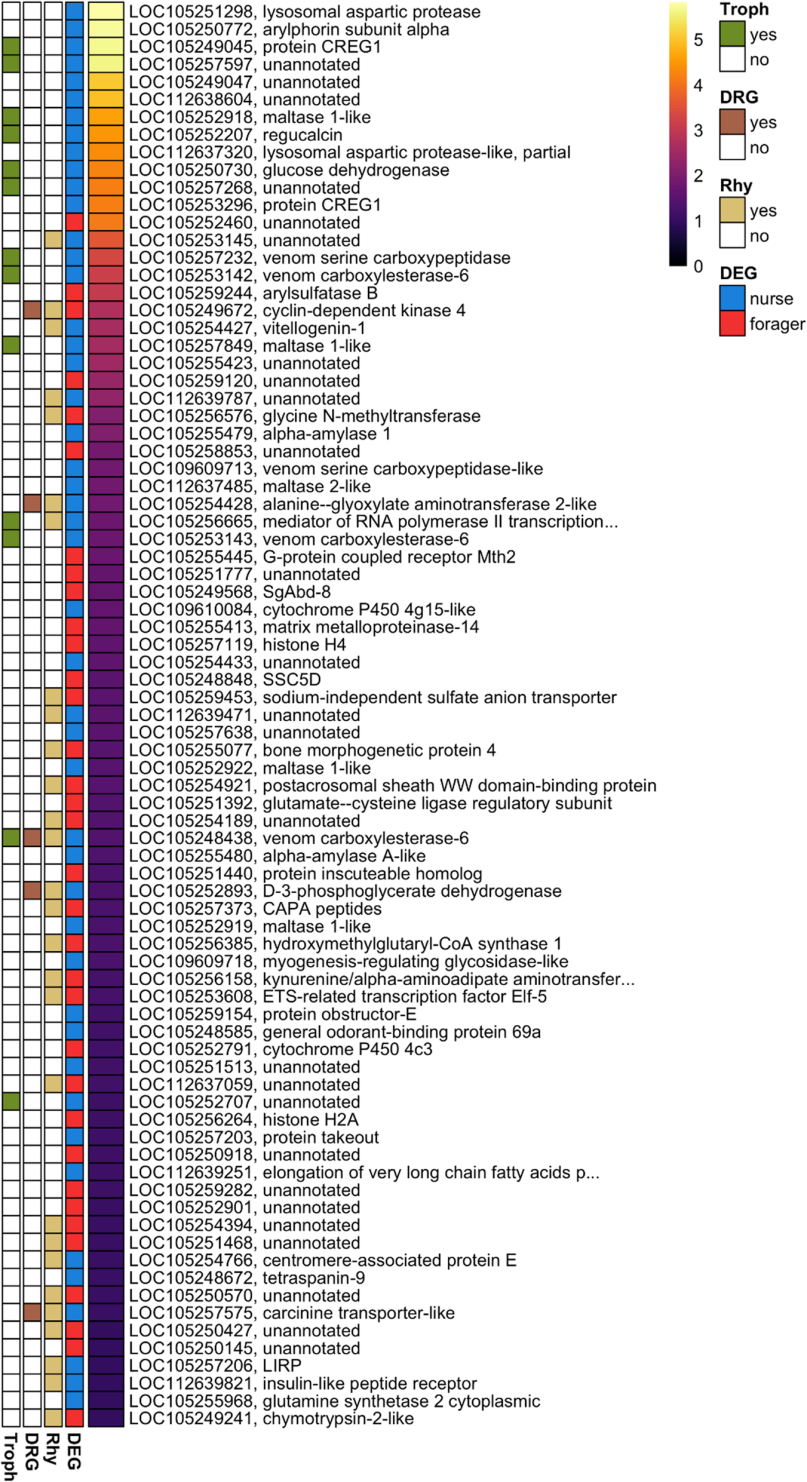
Differentially expressed genes between forager and nurse ant brains**.** Heatmap showing absolute (abs) log2-Fold-Change (log_2_FC) values for all 81 DEGs (q < 0.05 and abs(log_2_FC) ≥ 1), ordered from highest to lowest fold-change. The DEG column indicates if the gene is significantly higher expressed in foragers (red) or nurses (blue). For each DEG, the *C. floridanus* gene numbers and their blast annotations are provided. Genes with no blast annotation or annotated as uncharacterized protein are indicated as “unannotated”. The Rhy (rhythmic) column indicates genes that are significantly rhythmic in at least one of the ant castes. The DRG column indicates genes that are significantly rhythmic in both castes but oscillating at different periodicities. Genes that code for proteins previously found in the trophallactic fluid of *C. floridanus* are indicated in the Troph column

Past research has identified several genes and pathways that could be underlying behavioral division of labor [77, 151–155]. However, the extent of clock control over these key elements has not been explored yet. As such, we identified genes that were differentially expressed between the two ant castes throughout the day and determined if these differentially expressed genes (DEGs) showed any diurnal or ultradian oscillations. Of the 10,038 expressed genes in the brains of *C. floridanus*, only 81 were significantly differentially expressed between the two behavioral groups based on our stringent cut-off criteria (fold change ≥ 2, q-value < 0.05; Additional File 8, sheet 1). However, we should note that as many as 2439 genes displayed a fold-change greater than zero at 5% FDR (Additional File 8, sheet 1), which is consistent with the number of DEGs reported in prior studies that have compared gene expression in nurse and forager ants [77, 155]. Of these 81 DEGs, 34 were significantly higher expressed in forager brains, and the remaining 47 were higher expressed in nurses (Fig. 6; Additional File 8, sheet 1). The 34 genes that were higher expressed in foragers comprised of several genes with unidentified functions and did not contain any significantly enriched GO terms. In contrast, the 47 genes that were higher expressed in nurses contained five maltase and five alpha-amylase genes which resulted in a significant enrichment for the GO terms carbohydrate metabolic process and catalytic activity (Additional File 8, sheet 2). This suggests that nurses might be metabolically more active than foragers, which is in line with previous findings from another ant species [77].

Looking for oscillating genes among the DEGs that we identified in *C. floridanus*, we found that more than one-third (i.e., 28 of the 81 DEGs) were expressed rhythmically in either forager or nurse brains (Fig. 6). The set of 81 DEGs was significantly overrepresented in genes that show ultradian (8 h or 12 h) oscillations in daily expression (Odds-ratio: 2.18; p-value: 0.006). Of these clock-controlled DEGs, five genes oscillated at different periodicities in the two ant castes, providing further support for potential links between chronobiological and behavioral plasticity in *C. floridanus*. One of these differentially rhythmic genes, *Cyclin-dependent kinase 4* (*Cdk4*), was higher expressed and cycled every 12 h in forager brains, while it cycled with an overall lower expression in nurse brains every 24 h (Fig. 6, Additional File 8, sheet 1). The other four differentially rhythmic DEGs, *Alanine—glyoxylate aminotransferase 2-like* (*Agxt2*), *D-3-phosphoglycerate dehydrogenase* (*Phgdh*), *Carcinine transporter-like* (*CarT*) and *Venom carboxylesterase-6*, showed a higher overall expression in nurse brains where they cycled every 24 h, while foragers exhibited an 8 h oscillation in expression (Fig. 6, Additional File 8, sheet 1).

The results of our study, and previous findings with regards to *venom-carboxylesterase-6*, warrant speculation on the potential role of *venom-carboxylesterase-6* in mediating the links between chronobiological and behavioral plasticity. *Venom-carboxylesterase-6*, a gene that is both differentially expressed and differentially rhythmic in *C. floridanus* brains, is an abundant protein found in the trophallactic fluid of this species [71, 75]. In fact, we found that more than a quarter (13 out of 47) of all genes that were higher expressed in nurses encoded such orally transferred proteins, including all three copies of *venom-carboxylesterase-6* (Fig. 6). The protein encoded by *venom-carboxylesterase-6* is a JH esterase (JHE). JHEs are enzymes that degrade JH in insect hemolymph, thus, regulating JH titers and caste-associated behaviors in ants [75, 156]. The peak expression of the 24 h cycling *venom-carboxylesterase-6* in nurse brains was around ZT12-14, which corresponds to the peak time of colony foraging that we found in *C. floridanus* (Fig. 1, Additional File 4 and 6). As such, a *venom-carboxylesterase-6* mediated dip in JH levels could be contributing to a lower propensity of nurses to engage in extranidal tasks during peak colony foraging hours. In line with this reasoning, we found that the lowest dip in forager *venom-carboxylesterase-6* expression, and likely corresponding increased levels of JH, occur at ZT12, the onset of peak foraging activity (Fig. 1, Additional File 4 and 6). We should note that expression of trophallactic fluid genes in the brain is somewhat unexpected and that the expression of such genes could potentially result from remnant fat body cells during brain dissections. In our study, the expression levels of trophallactic fluid genes showed consistent differences between the two castes throughout the 24 h-day. Therefore, we suspect that the results are not an artefact of our dissections because they were conducted in the same way for the two castes. One would expect a more random distribution of trophallactic signals in our dataset if they were due to fat body cell contamination. However, if the signal originates in the brain or fat body cells remains unclear at this time and future studies using time-course single-cell RNA-Seq could be used to uncover tissue-specific daily expression profiles.

Even though not much is known about the role of circadian clocks in regulating behavioral plasticity in ants, previous studies have identified several genes and protein products that seem to be central regulators of behavioral plasticity in social insects [157]. Caste-specific differences in larval storage proteins, especially Vitellogenin (Vg) and Arylphorin subunit alpha, and JH have been consistently found across social insects. In bees, for example, high *Vg* levels and low JH titers correlate with nurse-like behaviors [158], whereas downregulation of *Vg* results in increased JH titers and a behavioral state characteristic of forager bees [159]. Similarly, nurses of the fire ant *Solenopsis invicta* show significantly higher *Arylphorin subunit alpha* expression as compared to the foragers [160]. Consistent with these previous findings, we found *C. floridanus* nurse brains to have significantly higher *Arylphorin-subunit-alpha* (50-fold) and *Vg* (sixfold) expression as compared to foragers (Fig. 6, Additional File 8, sheet 1). Additionally, our data showed that *Vg* expression is significantly oscillating every 24 h in nurse brains. Although not significant, *Arylphorin subunit alpha* also showed a *Vg*-like oscillatory expression in nurse brains (tau: 24 h, *p*: 0.09) (Additional File 5). However, forager brains showed no such rhythms in *Vg* or *Arylphorin subunit alpha* expression. As such, our study provides further support for a role of *Vg* and *Arylphorin subunit alpha* in behavioral division of labor and highlights a putative clock-control of these genes in nurse brains (Fig. 4). The functional role, if any, of a rhythmic *Vg* expression in ant physiology or behavior remains to be explored.

## Conclusion

The study presented here is providing a first look at the clock-controlled pathways in ants that could underlie caste-associated behavioral plasticity and sheds new light on the links between molecular timekeeping and behavioral division of labor in social insects. Understanding how an ant’s biological clock can predictably interact with its environment to produce distinct, yet stable, caste-associated chronotypes, lays the foundation for further molecular investigations into the role of biological clocks in regulating polyphenism in ant societies.

To produce high-interval time course data that reflects the transcriptional differences between forager and nurse ants throughout a 24 h day, we used an experimental setup that allowed us to reliably sample each ant caste and obtain their diurnal brain transcriptomes. The colony activity data that we collected had high enough resolution to even identify how the colony is able to quickly get back on track with regards to food collection efforts after a disturbance. More importantly, we found a reduced circadian time keeping in nurses as compared to foragers. This was evidenced by the vastly different number of genes that oscillated every 24 h in each ant caste, and the temporal segregation of clock-controlled processes, which is detectable in both castes but to a lesser extent in nurses. Our findings are, therefore, in line with the results of a previous study done in honeybees, which indicates that a difference in 24 h-rhythmic gene repertoire between foragers and nurses could be a more general phenomenon within eusocial Hymenoptera, and likely contributes to the caste-specific differences observed in behavioral activity rhythms.

Moreover, many genes that showed a diurnal expression in forager brains were expressed in an ultradian manner in nurses, instead of being entirely arrhythmic. Among the differentially rhythmic genes were essential components of the core and auxiliary feedback loops that form the endogenous clock of insects, as well as genes involved in metabolism, cellular communication and protein modification (Fig. 4). The ability of core clock and clock-controlled genes to oscillate at different harmonics of the circadian rhythm, and to switch oscillations from one periodicity to the other due to age or colony demands, might explain why chronotypes associated with ant behavioral castes are stable in undisturbed conditions, yet highly plastic and responsive to changes in their social context. However, it remains to be seen if the caste-associated differential rhythmicity that we observed is a general phenomenon across ant and other eusocial societies, or a species-specific trait. In addition, the potential for an actual adaptive function for maintaining both diurnal and ultradian rhythms in ant colonies will have to be further explored.

Finally, we found that the genes differentially expressed between forager and nurse brains are enriched in genes that show ultradian rhythms (periodicity: 8 h or 12 h). Additionally, several of these differentially expressed genes showed robust 24 h rhythms in nurse brains, including known regulators of JH titers in insects: *Vg* and *venom-carboxylesterase-6* (Fig. 4). Given the central role of Vg and JH in regulating division of labor in social insects, we propose that a mechanistic link between plasticity of the circadian clock and division of labor likely exists.

## Methods

### *Camponotus floridanus* collection and husbandry

Our study aimed to investigate daily gene expression differences in the brains of foragers and nurses. To prevent potential inter-colony variation in the degree of division of labor [161–163] from obscuring inter-caste differences, we used a single colony of *C. floridanus*. We collected a queen-absent colony of *C. floridanus* containing several thousand workers and abundant brood (eggs, larvae and pupa) from the University of Central Florida Arboretum in late April of 2019. This colony represents a typical medium-sized *C. floridanus* colony [164] that allowed us to study division of labor in an ecologically-relevant manner since (1) queenless colonies of *C. floridanus* as small as < 50 individuals already demonstrate forager-nurse caste differentiation [49], and (2) post-collection, we used this colony for experimentation as quickly as possible (i.e., within three weeks) to minimize any potential effects of queen-absence on overall colony behavior. Upon collection, we housed the colony in a fluon coated (BioQuip) plastic box (dimensions 42 × 29 cm, Rubbermaid) with a layer of damp plaster (Plaster of Paris) covering the bottom. We provided 15% sugar solution and water ad libitum and fed crickets to the colony every 2–3 days. We also provided the colony with multiple light-impervious, humid test-tube chambers (50 mL, Fisher Scientific) which they readily moved their brood into and used as a nest. Until the start of the experiment, we kept the colony in this setup inside a climate-controlled incubator (I36VL, Percival) at 25ºC, 75% relative humidity (rH), and a 12 h:12 h light–dark (LD) cycle.

### Experimental setup and timeline

To allow for visible behavioral division of labor between morphologically indistinguishable forager and nurse ant castes (see definitions below), we built a formicarium consisting of a nest box and a foraging arena (42 × 29 cm each, Rubbermaid). Both boxes had a layer of damp plaster covering the bottom. We carved multiple grooves into the plaster of the nest box to imitate nest chambers and kept the box covered at all times to ensure completely dark conditions. We placed the nest in a temperature-controlled darkroom at constant temperature and humidity (25ºC, 70% rH). The foraging arena was placed inside a climate-controlled incubator (I36VL, Percival) under a 12 h:12 h LD cycle without twilight cues. Lights ramped from zero to > 2000 lx within a minute when lights were turned on at Zeitgeber Time, ZT24 (or, ZT0, which indicates the same time of day) and turned off within the same short time at ZT12 (Additional File 9). We maintained constant temperature (25ºC) and humidity (75% rH) inside the incubator to ensure that the LD cycle was the primary rhythmically occurring cue, i.e., Zeitgeber, for circadian entrainment (Additional File 9). Abiotic factors in the foraging arena and nest box were monitored using HOBO data loggers (model U12, Onset) that logged light levels, temperature and humidity at 30 s intervals (Additional File 9). Food was provided ad libitum on an elevated circular feeding stage in the foraging arena to distinguish active feeding bouts from general extranidal visits (Additional File 10A). Feeders were replenished, and fresh frozen crickets were provided, every day between ZT2 and ZT4, throughout the experiment. The nest box was connected to the foraging arena with a 1.5 m long plastic tube (i.e., *Tunnel*, Additional File 10A), which allowed ants to visit to the foraging arena at any time of the day.

Once the formicarium was set up, we transferred the entire colony along with brood into the foraging arena. To incentivize the colony to move their brood into the dark nest box, we kept the foraging arena under constant light for three consecutive days. This also aided in the resetting of their biological clocks to allow for synchronized entrainment to the 12 h:12 h LD cycle. After 5 days of initial entrainment, we identified and marked foragers for three consecutive days (Day 6–8, Fig. 1, see below for details on mark-and-recapture). This was followed by another four days of entrainment (Pre-sampling entrainment, Day 9–12, Fig. 1) before we sampled nurse and forager ants at two-hour intervals, spanning an entire LD cycle on day 13 (see below for sampling details).

### Colony activity monitoring

The extranidal or outside nest activity of the colony (called *activity* from here on) was used as a proxy for detecting rhythmicity in colony behavior. Before sampling ants for RNASeq, we analyzed the activity data to (a) confirm colony entrainment to the LD cycle, (b) identify peak activity hours for forager identification and painting, and (c) confirm pre-sampling entrainment after foragers had been marked. We monitored colony activity during the entire experimental period by recording time-lapse videos of the foraging arena using a modified infra-red enabled camera (GoPro Hero 6) at 4 K resolution, set to capture one frame every 30 s at a wide field of view. To facilitate night-time recording, we installed a low intensity near-infrared light (850 nm, CMVision YY-IR30) above the foraging arena. We quantified extranidal activity throughout the experiment by counting the number of ants in the foraging arena on the feeding stage (feeding activity) and off the feeding stage (foraging activity) at one-hour intervals. The activity data can be found in Additional File 11.

### Identification of* Camponotus floridanus* behavioral castes

To measure and compare their daily rhythms in gene expression in forager and nurse brains, we sampled these behaviorally distinct castes using an approach similar to recent work that aimed to measure their trophallactic fluid protein levels [165]. We defined foragers as individuals that perform outside-nest (extranidal) tasks, including foraging for food. To identify foragers, we used a mark and recapture strategy. For three consecutive nights (Day 6–8, Fig. 1), we collected ants from the foraging arena during peak hours of extranidal activity (ZT13 to ZT16) as well as during relative dawn (ZT23 to ZT24). We marked new captures with a dab of white paint (Testors Enamel Paint) on their abdomen. Recaptures were marked with a second dab of white paint on their thorax. After painting, the ants were released back into the foraging arena. Previous studies have shown that such mark-recapture efforts can be used to successfully identify reoccurring foragers [166] and estimate forager abundance in ant colonies [167, 168]. Since peak foraging hours took place during the night-time, we installed a 660 nm red lightbulb (Byingo LED) in the darkroom and wore a red headlamp (Petzl Tikka) to provide us with enough visibility to perform the mark-recapture, while simultaneously minimally disturbing the ants. We identified and marked more than a hundred foragers at the end of the three-day forager identification phase (109 doubly marked, and 39 singly marked). Post forager identification, the whole colony was left undisturbed and allowed to recover from potential stress for four consecutive days of pre-sampling entrainment, prior to sampling ants for RNASeq.

We defined nurses as ants that remained inside the dark nest chambers (intranidal) and cared for brood*.* Extranidal workers, defined as foragers in this study, do not usually tend brood or frequent brood piles since ant colonies spatially organize themselves to reduce contact between foraging individuals and brood [169, 170]. Additionally, such proximity networks are stable over time and do not change in the absence of a queen [171]. As such, we identified nurses as unmarked individuals in the colony that were unlikely to have gone outside the nest and were in direct contact with the brood. To confirm that the bulk of brood care inside the nest was performed by unmarked ants, and not marked foragers, we performed qualitative intermittent behavioral observations for a total of 1–2 h per day during the pre-sampling entrainment period that followed mark-recapture (Days 9–11, Fig. 1). We observed the nest chambers under the same red light (660 nm) that illuminated the darkroom. Monitoring behavior inside the nest confirmed that marked “foragers” were less likely to be in direct contact with the brood (i.e., walking on the brood pile or grooming brood) and were not seen to be involved in brood relocation within the nest chambers. As such, we identified nurses as “unmarked” individuals found in direct contact with the brood or involved in brood care including relocation.

### Ant sampling and brain dissections

After identifying foragers and nurses and 12 days of colony entrainment to the 12 h:12 h LD, we collected ants for RNASeq under the same light–dark regime. We sampled ants from the colony every 2 h over a 24-h period, starting two hours after lights were turned on (ZT2) (Additional File 10B). At each sampling time point, we collected three foragers and three nurses from the colony and transferred them into individually labelled cryotubes (USA Scientific) for immediate flash freezing in liquid nitrogen. The whole process, from collection to flash freezing, took less than 60 s per sampled ant. Since *C. floridanus* foraging activity is predominantly nocturnal, we sampled foragers from inside the dark nest box during the light phase, and from the foraging arena during the dark phase (Additional File 10B). Nurses were always collected from inside the nest box. For sampling under dark conditions, we used the same intensity red-light as described for the mark-recapture and behavioral observations described above. Using this sampling regime, we collected 72 ants, which were stored at -80ºC until brain dissection.

To compare transcriptome-wide daily gene expression patterns in the brain tissue of foragers and nurses, we performed brain dissections of individual flash-frozen ants in ice-cold Hanks’ balanced salt solution (HBSS) buffer under a dissecting microscope. Prior to dissection, we removed both the antennae and pinned down the head of the ant using a pair of sharp forceps inserted into the antennal “sockets”. Next, using small scissors we made an incision around the head and removed the head capsule using another pair of forceps to expose the intact brain. Finally, we carefully extracted the brain from the head and removed any remains of other tissues attached to the brain. This clean, dissected brain was quickly transferred into a cryotube (USA Scientific) kept on dry ice. To preserve RNA integrity and quality of the ant brains, we performed all the above steps as swiftly as possible: brain dissections of individual foragers took an average of 4.6 (± 0.7) mins, whereas for a nurse it took 4.5 (± 0.5) mins. For each behavioral caste, at each sampling time point, we pooled three individually dissected brain samples for RNA extraction and sequencing (Additional File 10C). Immediately after dissection of all three forager/nurse brains for each time point, the cryotube was transferred to and kept in liquid nitrogen while we dissected the remaining ant brains. The resulting 24 samples were again stored at -80ºC until RNA extraction and library preparation. This sampling approach was designed to adhere to current recommendations for genome-wide time course studies using non-model systems [62, 172]. By pooling triplicates, we have accounted for intra-colony variation while still being able to choose a high sampling frequency (every 2 h) and read depth per sample (≥ 20 M per sample, see below) in order to maximize accurate detection of the majority of cycling transcripts in *C. floridanus* brains [172].

### RNA extraction, library preparation and RNASeq

To obtain time course transcriptomes for each of the behavioral castes, we extracted total RNA to prepare sequencing libraries for Illumina short-read sequencing. Two frozen steel ball bearings (5/32″ type 2B, grade 300, Wheels Manufacturing) were added to each cryotube containing the pooled brain tissues to homogenize them using a 1600 MiniG tissue homogenizer (SPEX) at 1300 rpm for 30 s while keeping the samples frozen. We isolated total RNA from the disrupted, frozen brain tissues by dissolving the material into Trizol (Ambion) followed by a wash with chloroform (Sigma) and a purification step using RNeasy MinElute Cleanup columns and buffers (Qiagen) [173]. For each library preparation, we used 500 ng total RNA to extract mRNA with poly-A magnetic beads (NEB) and converted this mRNA to 280–300 bp cDNA fragments using the Ultra II Directional Kit (NEB). Unique sequencing adapters were added to each cDNA library for multiplexing (NEB). The quantity of extracted RNA and cDNA libraries were measured using Qubit (Invitrogen), whereas the quality and integrity were assessed using an Agilent Tapestation. All twenty-four cDNA libraries were sequenced as 50 bp single-end reads using two lanes on an Illumina HiSeq1500 at the Laboratory for Functional Genome Analysis (Ludwig-Maximilians-Universitat Gene Center, Munich). Read data are available under BioProject PRJNA704762. After sequencing, we removed sequencing adapters and low-quality reads from our RNASeq data with BBDuk [174] as a plug-in in Geneious (parameters: right end-low quality trim, minimum 20; trim both ends—minimum length 25 bp) (Biomatters). Post-trimming, we retained an average of 22 million reads per sample, which is well beyond the minimal read depth sufficient to identify the majority of high amplitude 24 h-rhythmic transcripts in insects [172]. Subsequently, we used HISAT2 [175] to map transcripts to the latest Cflo v7.5 genome [72], followed by normalizing each sample to Fragments Per Kilobase of transcript per Million (FPKM) with Cuffdiff [176].

### Data analyses

We confirmed daily rhythms in colony activity with the WaveletComp package [76]. Using wavelet analyses, we investigated the extranidal activity of foragers for the presence of 24 h-rhythms in colony behavior, the potential presence of ultradian rhythms, and to infer synchronicity between the number of ants actively feeding or present on the feeding stage (feeding activity), and those present in the remainder of the foraging arena (foraging activity).

We used the rhythmicity detection algorithm empirical JTK-Cycle (eJTK) [78, 79] to test for significant diurnal and ultradian rhythms in gene expression in foragers and nurses using waveforms of period lengths (tau) equal to 24 h, 12 h and 8 h. The algorithm, eJTK, builds on the non-parametric JTK-Cycle [177] by allowing detection of asymmetric sinusoidal waveforms since there is no a priori reason to assume that biological rhythms are symmetric [78]. Furthermore, a recent comparative analysis of different rhythmicity detection algorithms suggests that eJTK is a highly robust method for detection of rhythmic features [63]. Only genes that had diel expression values ≥ 1 FPKM for at least half of all sampled timepoints were tested for rhythmicity. For a set period length, a gene was considered to be significantly rhythmic if it had a Gamma p-value < 0.05. To test if certain genes could be clustered together based on similar temporal peak activity, we used an agglomerative hierarchical clustering framework (method: complete linkage) using the ‘hclust’ function in the ‘stats’ package for R.

Time-course sampling of foragers and nurses enabled us to account for diel fluctuations in expression levels when identifying genes that were differentially expressed between the two ant groups throughout the day (i.e., DEGs). To determine differentially expressed genes, we used the linear modelling framework proposed in LimoRhyde [178], but without an interaction between treatment and time. A gene was considered differentially expressed if treatment was found to be a significant predictor (at 5% FDR) and the difference in mean diel expression between foragers and nurses was at least twofold (i.e., abs(log_2_-fold-change) ≥ 1). The more stringent twofold-change threshold allowed us to investigate the putative clock-control of only those genes that were more likely to have a biologically relevant difference in gene expression between forager and nurse brains. LimoRhyde is generally used to test if genes of the same periodicity are differentially rhythmic in phase or amplitude, inferred from a significant interaction between treatment and time. However, we did not find significant differences in phase or amplitude for any of the genes that were found to have 24 h rhythms in both foragers and nurses (Additional File 12). Therefore, we indicated a gene as differentially rhythmic (i.e., DRGs) if it significantly cycled in both ant castes but with different period lengths.

To perform functional enrichment analyses of significant gene sets, we wrote a customized function that performs a hypergeometric test through the *dhyper* function in R. The code is available on GitHub (https://github.com/debekkerlab/Will_et_al_2020). The function takes the following inputs: (1) user-provided geneset to test enrichment on, (2) user-provided background geneset to test enrichment against, and (3) functional gene annotations (e.g., GO terms) to test enrichment for. Among other things, the function outputs a Benjamini Hochberg-corrected p-value for each annotation term to indicate if it is significantly enriched in the test geneset. We used all genes that were found to be “expressed” (≥ 1 FPKM expression for at least one sample) in the brains of foragers or nurses as the background geneset for functional enrichment tests. To analyze the functional enrichment of Gene Ontology (GO) predictions, we used the GO term annotations [73] for the most recent *C. floridanus* genome (v 7.5) [72]. We only tested terms annotated for at least 5 protein coding genes and significance was inferred at 5% FDR.

Homologs of known core-clock genes (*cgs*) and clock-modulator genes (*cmgs*) *in C. floridanus* were identified using previously published hidden-markov-models (HMMs) for well-characterized clock proteins of two model organisms: *Drosophila melanogaster* and *Mus musculus* [179]. We used *hmmersearch* to query these HMM profiles against the entire *C. floridanus* proteome (Cflo_v7.5) [72] with default parameters (HMMER v3.2.1 [180]). To identify orthologs shared between *C. floridanus* and flies, mammals or honey bees we used proteinortho5 [181].

All data wrangling, statistical tests and graphical visualizations were performed in RStudio [182] using the R programming language v3.5.1 [183]. Heatmaps were generated using the pheatmap [184] and viridis [185] packages. Upset diagrams were used to visualize intersecting gene sets using the UpsetR package [186]. We used a Fisher’s exact test for identifying if two genesets showed significant overlap using the GeneOverlap package [187].

## Supplementary Information

**Additional file 1. **

**Additional file 2.**

**Additional file 3.**

**Additional file 4.**

**Additional file 5.**

**Additional file 6.**

**Additional file 7.**

**Additional file 8.**

**Additional file 9.**

**Additional file 10.**

**Additional file 11.**

**Additional file 12.**

## Data Availability

Raw sequencing reads generated for this study have been deposited in NCBI under BioProject PRJNA704762 (https://www.ncbi.nlm.nih.gov/bioproject/PRJNA704762). The datasets supporting the conclusions of this article are included within the article and its additional files. Data analysis and visualization for this study was done using code written in R, Python and Bash, and can be found through GitHub (https://github.com/debekkerlab/Das_et_al_2021). Additionally, an RSQLite database containing all processed data can be provided upon request.

## Abbreviations

DEGs: Differentially expressed genes
DRGs: Differentially rhythmic genes
eJTK: Empirical JTK Cycle
for-8 h: 8H rhythmic in foragers
for-12 h: 12H rhythmic in foragers
for-24 h: 24H rhythmic in foragers
FPKM: Fragments per kilobase of transcripts per million mapped reads
GO: Gene ontology
LD: Light–dark
nur-8 h: 8H rhythmic in nurses
nur-12 h: 12H rhythmic in nurses
nur-24 h: 24H rhythmic in nurses
ZT: Zeitgeber time
TTFL: Transcription-translation feedback loop
