# Supplementary figures and images for: Time-course RNASeq of Camponotus floridanus forager and nurse ant brains indicate links between plasticity in the biological clock and behavioral division of labor
Source: BMC Genomics. 2022 Jan 15;23:57. doi: 10.1186/s12864-021-08282-x (PMC8760764; doi:10.1186/s12864-021-08282-x)

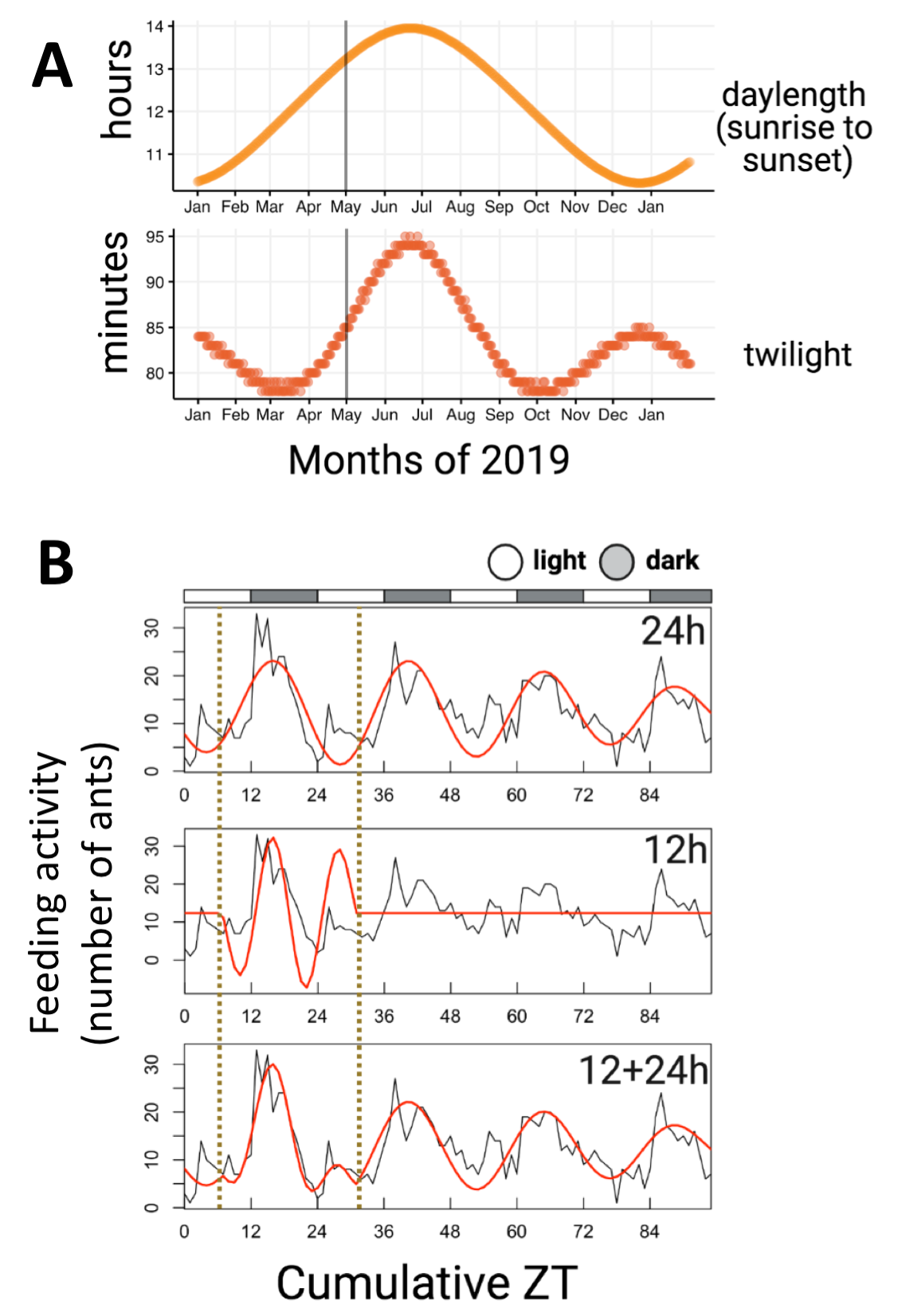

Supplement: Supplementary file 1 — Additional file 1. [file 12864_2021_8282_MOESM1_ESM.png]

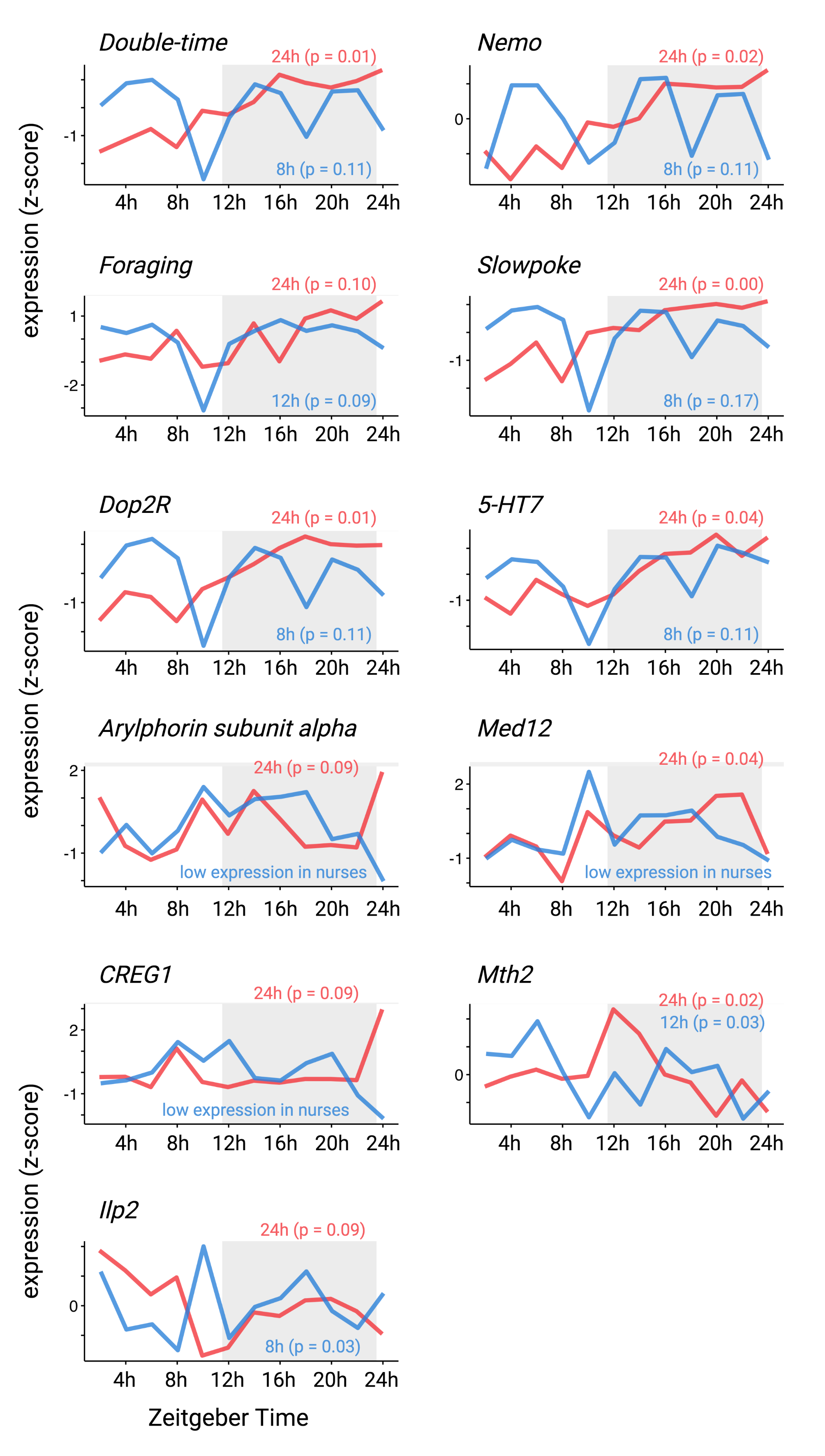

Supplement: Supplementary file 5 — Additional file 5. [file 12864_2021_8282_MOESM5_ESM.png]

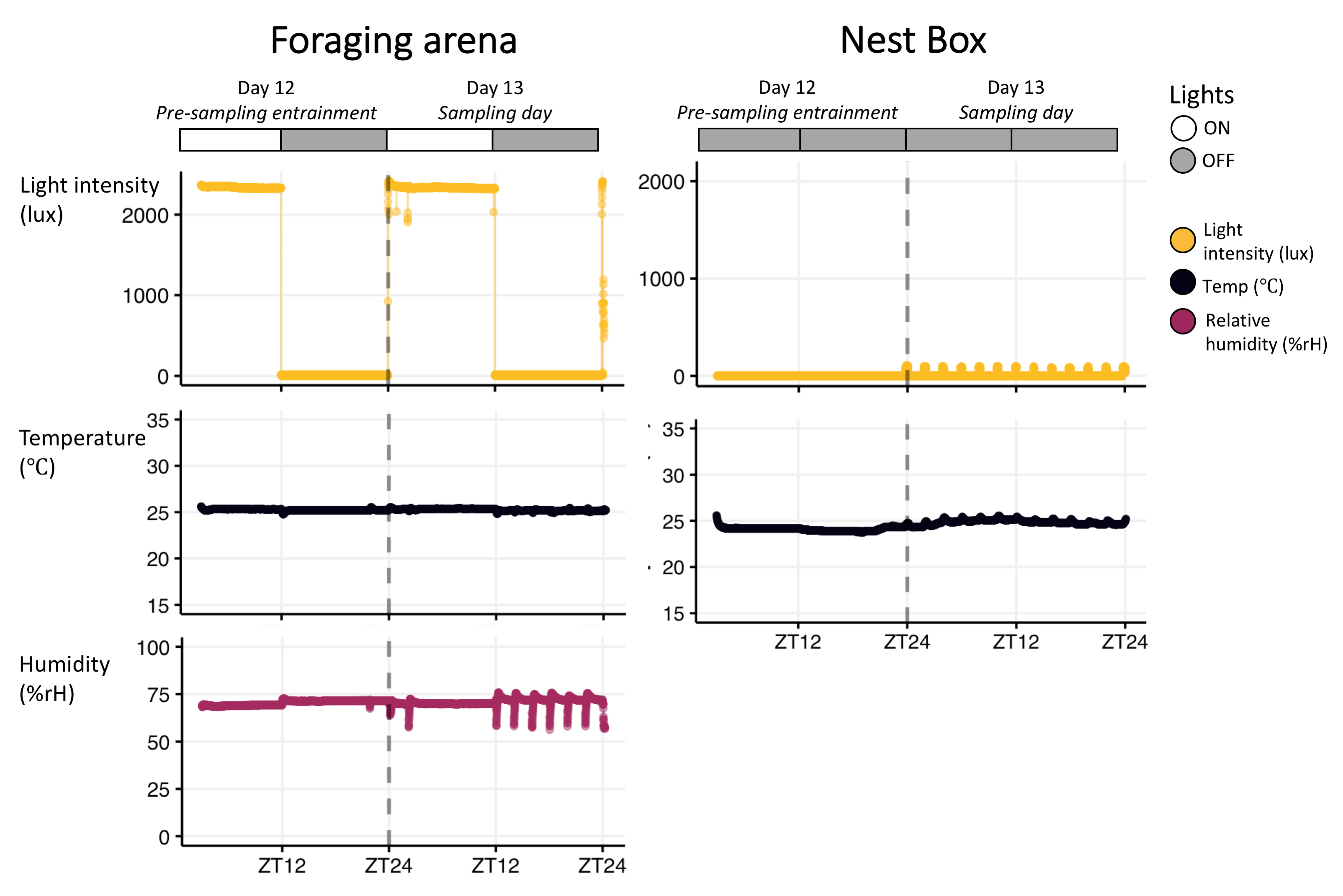

Supplement: Supplementary file 9 — Additional file 9. [file 12864_2021_8282_MOESM9_ESM.png]

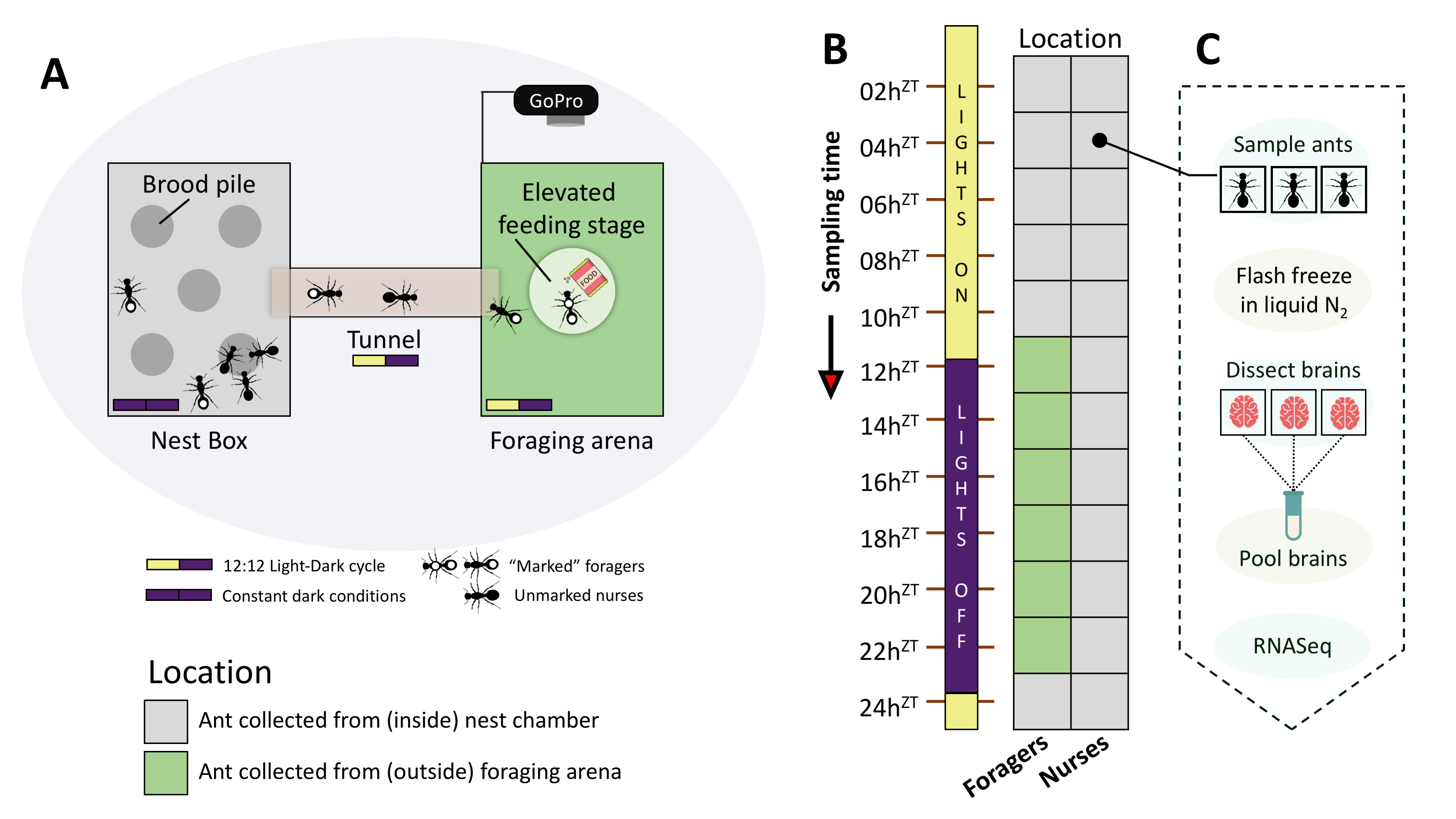

Supplement: Supplementary file 10 — Additional file 10. [file 12864_2021_8282_MOESM10_ESM.png]
